# Footprints of microRNAs in Cancer Biology

**DOI:** 10.3390/biomedicines9101494

**Published:** 2021-10-19

**Authors:** Yaashini Rajasegaran, Adam Azlan, Aliaa Arina Rosli, Mot Yee Yik, Khor Kang Zi, Narazah Mohd Yusoff, Emmanuel Jairaj Moses

**Affiliations:** Advanced Medical and Dental Institute, Universiti Sains Malaysia, Bertam, Kepala Batas 13200, Pulau Pinang, Malaysia; yaashini@student.usm.my (Y.R.); adamazlan@student.usm.my (A.A.); aliaarosli@student.usm.my (A.A.R.); yymot81@gmail.com (M.Y.Y.); kangzi@student.usm.my (K.K.Z.); narazah@usm.my (N.M.Y.)

**Keywords:** microRNA (miRNA), cancer biology, gene regulation, biomarkers, therapeutic targets

## Abstract

MicroRNAs (miRNAs) are short non-coding RNAs involved in post-transcriptional gene regulation. Over the past years, various studies have demonstrated the role of aberrant miRNA expression in the onset of cancer. The mechanisms by which miRNA exerts its cancer-promoting or inhibitory effects are apparent through the various cancer hallmarks, which include selective proliferative advantage, altered stress response, vascularization, invasion and metastasis, metabolic rewiring, the tumor microenvironment and immune modulation; therefore, this review aims to highlight the association between miRNAs and the various cancer hallmarks by dissecting the mechanisms of miRNA regulation in each hallmark separately. It is hoped that the information presented herein will provide further insights regarding the role of cancer and serve as a guideline to evaluate the potential of microRNAs to be utilized as biomarkers and therapeutic targets on a larger scale in cancer research.

## 1. Introduction

The hallmarks of cancer were initially proposed by Douglas Hanahan and Robert Weinberg to organize the principles that provide a logical framework for understanding the cellular mechanisms of oncogenesis. These hallmarks were classified into six major categories and had been very influential in cancer research [1]. After about a decade of intense research regarding the fundamentals of cancer biology, a revision regarding the classification was proposed to incorporate the current knowledge of cancer development [2]. The organization of the hallmarks of cancer allowed researchers to understand the core traits of cancer, regardless of the origins of the cancer cells. As our understanding of cancer improved over the past two decades, updated lists of cancer hallmarks have been produced, culminating in the most recent review by Fouad and Aanei in 2017 titled “Revisiting the Hallmarks of Cancer”. The distinct hallmarks are selective proliferative advantage, altered stress response, vascularization, invasion and metastasis, metabolic rewiring, abetting microenvironment and immune modulation [3].

Numerous studies have reported the involvement of miRNAs in various human cancers [4,5,6]. This comes as no surprise, as microRNAs (miRNA) have been known to play pivotal roles in many major cellular functions, such as development, differentiation, growth and metabolism [7]. MiRNAs are small, highly conserved non-coding RNAs with an average length of 22 nucleotides that are primarily involved in the regulation of gene expression. These miRNAs may either assume an oncogenic role or tumor-suppressive role in cancer development, depending on the target gene [8,9]. Given the indispensable role of miRNAs in cancer, an in-depth description regarding the involvement of microRNAs (miRNAs) in each cancer hallmark will be presented in this review.

## 2. Hallmark 1: Selective Proliferative Advantage

One of the defining characteristics of cancer cells is their ability to grow and proliferate uncontrollably compared to normal cells, which leads us to the first hallmark of cancer—selective growth and proliferative advantage. Cell growth and proliferation is a tightly regulated process in normal cells; however, alterations in this process allow the cancer cells to grow and proliferate uncontrollably [10]. This can be achieved via multiple pathways, for example growth ligands, growth receptors, cytosolic signaling and cell cycle regulation [11,12]. By altering these pathways, the cancer cells send signals that promote growth and proliferation while compromising signals that inhibit growth.

### 2.1. Intracellular Signal Pathways Dysregulation in Cancer Cells

**Figure 1:**
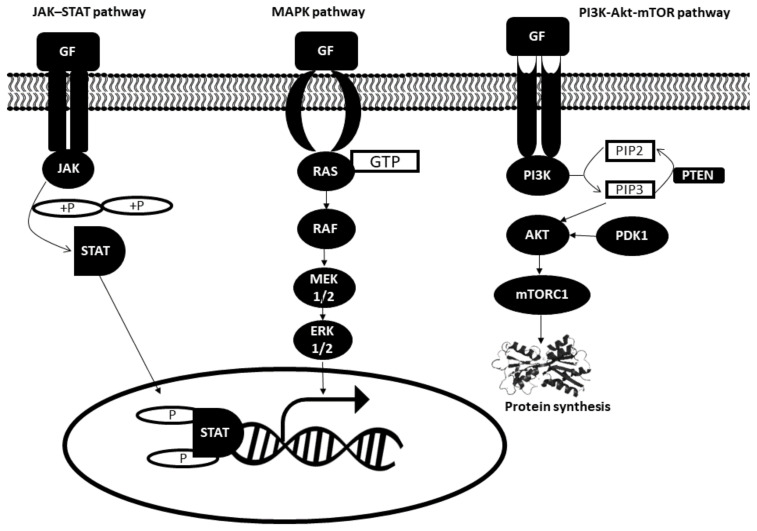
Main players involved in intracellular signalling pathways. GF: Growth factor; JAK: Janus tyrosine kinase; STAT: Signal transducer and activator of transcription proteins; MAPK: Mitogen-activated protein kinase; MEK: Mitogen-activated protein kinase; ERK: Extracellular-signal-regulated kinase; PI3K: Phosphatidylinositol 3 kinase; mTOR: Mammalian target of rapamycin; PDK1: Phosphoinositide-dependent kinase 1; PIP2: phosphatidylinositol 4,5-biphosphate; PIP3: phosphatidylinositol 3,4,5-triphosphate; PTEN: phosphatase and tensin homolog; P: Phosphate group.

Binding of growth factors to receptors on the surface of the cell activates a series of intracellular signal networks namely JAK–STAT, the mitogen-activated protein (MAP) kinase and the phosphatidylinositol 3 (PI3) kinase pathways. Disruption in any one of these pathways may give rise to defective mitogenic signaling. All three pathways are illustrated in Figure 1 and further explained below [13,14,15,16].

#### 2.1.1. JAK–STAT Pathway

The JAK–STAT pathway comprises three main components: tyrosine-kinase-related receptor, Janus tyrosine kinase (JAK) and signal transducer and activator of transcription proteins (STAT). JAK is a family of cytoplasmic proteins that aids in transducing extracellular signals to intracellular downstream cascades. Growth factor molecules bind to their respective receptors on the plasma membrane, which activates intracellular cytoplasmic JAKs that proceed to recruit STATs to the receptor. STATs are phosphorylated by JAKs and other cytosolic serine and threonine kinases, such as ERK1/2. This is followed by its translocation from the cytoplasm into the nucleus. STAT then binds to promoters of the target gene and activates gene transcription.

#### 2.1.2. MAPK Pathway

The mitogen-activated protein kinase (MAPK) pathway, also known as the Ras–Raf–MEK–ERK pathway, mediates cell proliferation. In response to the binding of growth factor to the receptor, inactive RAS (a GTPase) is converted to its active form. RAS activation is followed by RAF activation, which subsequently leads to the phosphorylation of downstream MEK1/2 followed by ERK1/2. Activated ERK1/2 then mediates the transcription of target genes such as c-MYC and others.

#### 2.1.3. Phosphatidylinositol 3 Kinase (PI3K) Pathway

PI3K-Akt-mTOR pathway is also involved in cell proliferation. Binding of growth factors to receptors located on the plasma membrane activates PI3K, which catalyzes the conversion of phosphatidylinositol 4,5-biphosphate (PIP2) to phosphatidylinositol 3,4,5-triphosphate (PIP3). PIP3 then executes its function by recruiting Akt to the membrane to be activated by phosphoinositide-dependent kinase 1 (PDK1). Akt activation then relieves the inhibition on mTORC1, which is mainly involved in protein synthesis. On the other hand, phosphatase and tensin homolog (PTEN) dephosphorylates PIP3 and impairs Akt activation.

### 2.2. Cell Cycle Dysregulation in Cancer Cells

**Figure 2:**
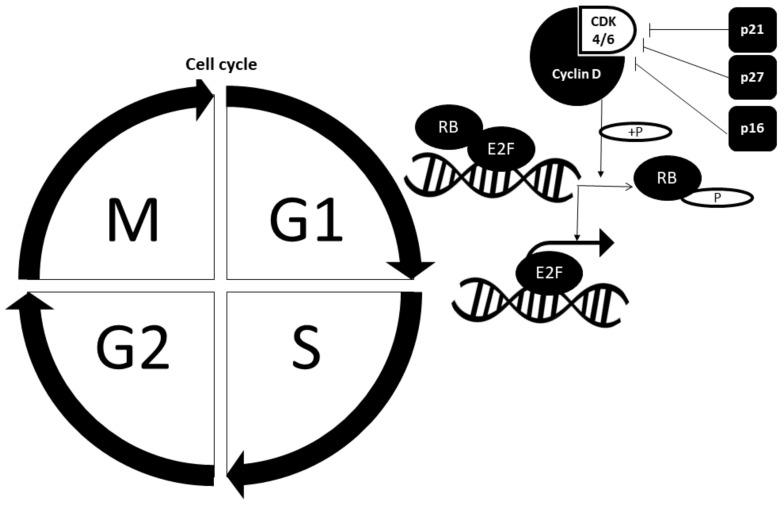
Regulation of cell cycle progression from G1 phase to S phase is controlled by various players. G_1_: Gap 1 phase; S: Synthesis phase; G_2_: Gap 2 phase: M: Mitotic phase; RB: Retinoblastoma; E2F: CDK: Cyclin-dependent kinase; P: Phosphate group.

Cell cycle is divided into the interphase (G_1_, S and G_2_ phase) and mitotic phase. The proliferative fate of a cell not only depends upon its entry into the cell cycle but also upon its complete progression through each phase. Various regulatory proteins act as gatekeepers of the cell cycle and deregulation often leads to formation of cancer. The regulatory proteins that are actively involved in safeguarding the cell cycle are retinoblastoma (RB) protein and cell cycle checkpoint proteins such as cyclin-dependent kinases (CDK), cyclins and cyclin-dependent kinase inhibitors (CDKi). Figure 2 illustrates the cell cycle regulated by crucial cell cycle checkpoint proteins.

#### 2.2.1. Retinoblastoma Pathway

The RB pathway comprises RB-CDK4/6-cyclin D. Its function is to control the cell cycle progression from the G_1_ phase to the S phase. Cyclin D binds to CDK4/6 to form a complex, and together they phosphorylate Rb and render Rb inactive. Hypo-phosphorylated Rb initially binds to E2F and repress its action. Nevertheless, Rb phosphorylation leads to release of E2F from transcriptional repression, which then allows for transcription of S-phase-promoting genes, thereby promoting cell progression from G1 to S phases [10].

#### 2.2.2. Cell Cycle Checkpoint Proteins

Cyclin and cyclin-dependent kinases always work in tandem. The most crucial cyclin–CDK complexes in the cell cycle are cyclin E-CDK2 and cyclin D-CDK4/6. Both complexes are associated with G1 phase to S phase progression. On the other hand, cyclin-dependent kinase inhibitors (CKI) contribute to abrogation of cell cycle progression mediated by growth-inhibitory signals. Examples of CKIs include p27, p21 and p16. Cell cycle progression from the S phase to G2 phase and mitotic phase is also tightly regulated by other cyclin–CDK complexes and various regulatory proteins [17].

### 2.3. The Role of miRNAs in Selective Proliferative Advantage

MiRNAs are involved in the regulation of proliferation-related signal pathways; miRNAs can exert either direct or indirect effects on proliferative signal pathways. Direct effects are mediated by directly targeting the 3′ untranslated region (3′UTR) of mRNA of genes that directly activate or inhibit cell proliferation. Indirect effects occur by targeting upstream or downstream regulators of genes that directly regulate cell proliferation.

MiRNAs are known to affect and dysregulate intracellular signal pathways. It was found that miR-101 directly targets the 3′UTR of MAPK kinase 1 (MEK1) mRNA, which is involved in the MAPK/ERK pathway. This results in attenuation of diffuse large B cell lymphoma (DLBL) cell proliferation [18]. Another miRNA that is implicated in the regulation of the PI3K/Akt pathway is miR-20a, which modulates its oncogenic actions in multiple myeloma by negatively regulating PTEN, a tumor suppressor that exerts its inhibitory effects on Akt activation and ultimately cell proliferation [19]. MiRNA may also disrupt the functions of cell cycle checkpoint kinases directly. Retinoblastoma (Rb), one of the crucial players in cell cycle progression, is a direct target of miR-590 in T cell acute lymphoblastic leukemia reported by Miao et al. [20]. Downregulation of Rb by miR-590 leads to an increase in cell proliferation. Table 1 summarizes other miRNAs involved in this hallmark with regards to their target, mechanisms of action and cancer types.

## 3. Hallmark 2: Altered Stress Response

The next hallmark is an altered stress response favoring overall survival. Cells have a variety of adaptations and responses to stress that damage the cells such as DNA repair, apoptosis, autophagy and senescence. Cells might be eliminated to maintain the overall health of the tissue under stressed conditions; however, cancer cells in their quest for continual growth and survival will alter their stress response [37]. Four types of stress response, namely DNA repair, apoptosis, autophagy and senescence, are highlighted below.

### 3.1. DNA Repair Pathways

The cells in our body are constantly faced with various genotoxic insults on a daily basis that can lead to DNA lesions. DNA lesions can either occur endogenously or exogenously (ultraviolet rays, ionizing radiation, genotoxic chemicals). These DNA lesions must be repaired promptly, as they may cause genomic instability and contribute to the overall survival of cells. DNA lesion repair is curated by the DNA damage response in our body. The typical DNA damage repair (DDR) includes: (1) sensing the damage; (2) checkpoint activation; (3) DNA damage repair.

Initiation of DDR starts with the formation of the DNA damage sensor MRN complex comprised of MRE11, Rad50 and NBS1 at the site of the lesions. Each of the players in this complex has its own distinct role. MRE11 (meiotic recombination 11 homolog) exhibits DNA exo- and endonuclease activity. Nijmegen breakage syndrome protein 1 (NBS1) recruits other DNA damage regulatory proteins to the lesion site, whereas Rad50 binds DNA ends together [38]. Binding of the MRN complex to the site of the lesion then activates and recruits ataxia–telangiectasia mutated (ATM) or ataxia–telangiectasia and rad3-related (ATR). ATM is normally recruited when there is a double-strand break (DSB), whereas ATR is recruited when there is a single-strand break (SSB) or replication stress [39]. BRCA1 is a key player in DNA repair machinery that is activated by both ATM and ATR [40]. H2AX, acting as DNA damage sensor, is recruited to the lesion site and is phosphorylated by ATM into ɣH2AX. Then, ɣH2AX recruits other DNA damage repair factors to initiate the repair process [41]. Poly(ADP-ribose) polymerase 1 (PARP1) is one of the first critical responders to DNA damage (DNA strand breaks), which acts by mounting the DNA repair mechanism accordingly [42].

Checkpoint activation is crucial for DNA repair processes. Activated ATR phosphorylates checkpoint kinase 1 (Chk1), which then goes on to bind to CDC25C, marking it for degradation by the ubiquitin pathway. This action results in inhibition of cell cycle progression. Chk1 also exerts its inhibitory action on cell cycle progression by activating p53. Chk2 is activated by ATM and shares the same function as Chk1 [43].

**Figure 3:**
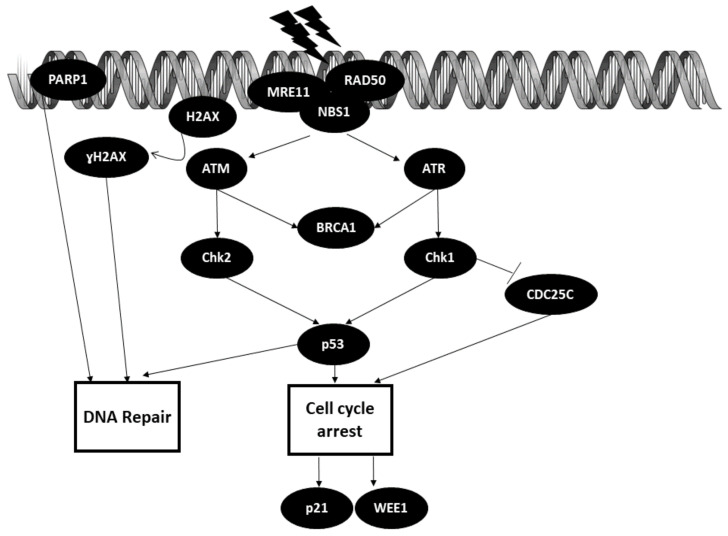
Crucial regulators of DNA damage repair (DDR) mechanism. PARP1: Poly (ADP-ribose) polymerase 1; MRE11: meiotic recombination 11 homolog; NBS1: Nijmegen breakage syndrome protein 1; ATM: Ataxia–telangiectasia mutated; H2AX: H2A histone family member X; ATR: Ataxia–telangiectasia and Rad3-related; BRCA1: Breast cancer 1; Chk2: Checkpoint kinase 2; Chk1: Checkpoint kinase 1.

DNA damage repair is based on the decision to undergo cell cycle arrest, DNA repair or apoptosis mediated by p53. P21 and WEE1 kinase are the downstream effectors of p53. P21 induces cell cycle arrest, whereas WEE1 prevents entry into the mitotic phase. As for DNA repair, p53 modulates the repair mechanism by regulating genes involved in DNA repair pathways, such as base excision repair, mismatch repair, nucleotide excision repair, translesion DNA synthesis (TLS), non-homologous end joining (NHEJ) and homologous recombination (HR) [44,45]. The DNA repair mechanism is illustrated in Figure 3.

#### The Role of miRNAs in DNA Repair Mechanisms

The miRNAs are able to regulate the DNA repair mechanism by targeting the players involved in the repair machinery via direct or indirect action. DNA damage repair involves vast networks that are interrelated, while miRNA regulation of any players in the network may affect the repair process, either positively or negatively. PARP1 is the direct target of miR-7-5p, leading to abrogation of DNA damage repair in small cell lung cancer as reported by Lai et al. [46]. The same finding was reported in cervical cancer by Yang et al. [47]. Furthermore, miR-203a-3p negatively regulates ATM in ovarian cancer cells, leading to cell cycle arrest [48]. Other examples of miRNAs that participate in DNA repair are listed in Table 2.

### 3.2. Autophagy

Autophagy is a cellular process defined as the breakdown of damaged proteins or organelles and recycling of the macromolecular components for the benefit of other metabolic processes in the cells. Dysregulation of autophagic pathways may either promote or inhibit tumor progression. Cancer cells may hijack the autophagy mechanism and use it to their advantage in ensuring overall survival [59].

The autophagy process is typically made up of five stages, namely initiation, vesicle nucleation, vesicle elongation, vesicle fusion and degradation, which take place in the cell cytoplasm. Autophagy may be initiated in response to nutrient starvation or any other signals. Under normal conditions, the mTORC1 complex inhibits the ULK1 complex, which sets off the autophagy cascade by inducing the formation of phagophore; however, in cases of nutrient starvation, mTORC1 complex inhibition of the ULK1 complex is released, initiating autophagy. The mTORC1 complex is made up of three components, namely mTOR, mLST8 and RAPTOR. The ULK1 complex is comprised of ULK1, ULK2, ATG13, ATG101 and FIP200.

The second stage includes the formation of Beclin1 complex induced by the ULK1 complex, which is made up of various autophagic proteins, such as BCL2 interacting protein (Beclin1), activating molecule in Beclin-1-regulated autophagy (AMBRA1), phosphatidylinositol 3-kinase catalytic subunit type 3 and regulatory subunit 4 (VPS34 and VPS15), UV radiation resistance-associated gene protein (UVRAG) and ATG14. Beclin1 complex formation can be negatively regulated by BCL-2 and BCL-X_L_ to inhibit autophagy.

Vesicle elongation is modulated by two ubiquitin-like conjugation systems that aid in the formation of autophagosomes. For the first conjugation system, ATG5 is conjugated to ATG12 with the help of enzymes such as ATG7 and ATG10. The ATG5-ATG12 conjugate binds to ATG16L1 to act as a facilitator to conjugate microtubule-associated protein 1A/1B LC3 (LC3-I) to phosphatidylethanolamine (PE). The second system involves conjugation of LC3-I with PE to form LC3-II mediated by ATG4B, ATG7 and ATG3. This stage ends with incorporation of LC3-II into the autophagosome membrane.

**Figure 4:**
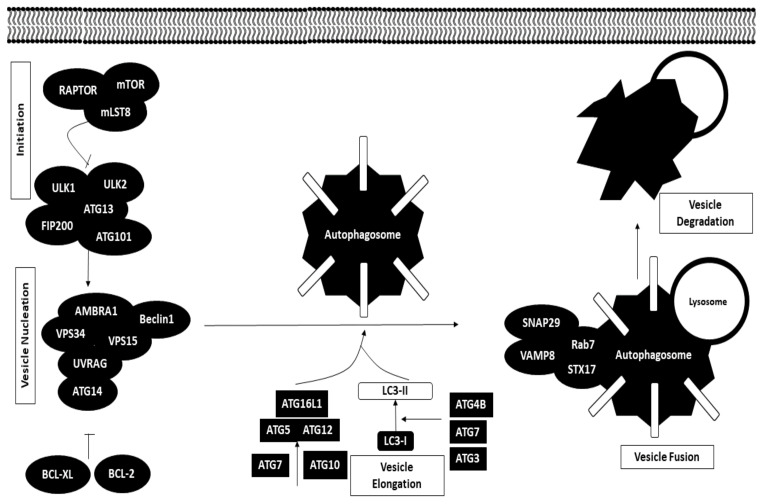
A brief overview of the autophagy process. RAPTOR: Regulatory-associated protein of mTOR; mTOR: Mammalian target of rapamycin; mLST8: mTOR-associated Protein, LST8 Homolog; ULK: Unc-51-like kinase; ATG: Autophagy-related gene; FIP200: FAK family kinase-interacting protein-200kD; AMBRA1: Autophagy And Beclin1 regulator 1; VPS34: phosphatidylinositol 3-kinase catalytic subunit type 3; VPS15: phosphatidylinositol 3-kinase regulatory subunit 4; UVRAG: UV radiation resistance-associated gene; BCL-xL: B cell lymphoma extra-large; BCL-2: B cell lymphoma 2; LC3: Microtubule-associated protein 1A/1B-light chain 3; SNAP29: Synaptosome-associated protein 29; VAMP8: Vesicle-associated membrane protein 8; STX17: syntaxin 17.

The fourth stage revolves around the fusion of autophagosome with lysosome mediated by SNARE proteins, including syntaxin-17 (STX17), synaptosome-associated protein 29 (SNAP29) and vesicle-associated membrane protein 8 (VAMP8), together with Rab7. Degradation of autophagosome contents is then carried out by the pH-sensitive enzymes in the lysosome [59,60]. The autophagy mechanism is illustrated in Figure 4.

#### The Role of miRNAs in Altering Autophagy Mechanisms

Autophagy can be regulated by miRNAs at various stages of autophagy signal pathways by exerting either direct or indirect actions. Direct autophagy-promoting action is seen when overexpression of miR-423-5p occurs in hepatocellular carcinoma following treatment with sorafenib, which increases the levels of ATG7 and LC3-II [61]. Indirect miRNA regulation can be seen in miR-423-5p, which induces autophagy by directly targeting Bcl-2-like protein 11 (Bim), a negative regulator of Beclin1, which is a crucial autophagy regulator in gastric cancer [62,63]. Regarding the autophagy-inhibiting miRNA, miR-409-5p targets and negatively regulates FIP200, which is crucial for assembly of the ULK1 initiation complex in ovarian cancer as reported by Cheng et al. [64]. Other examples of miRNA involved in autophagy are depicted in Table 3.

### 3.3. Apoptosis

Apoptosis is a type of cell death mechanism that is considered a normal biological process. It governs and maintains the balance between cell survival and cell death. A deranged apoptotic mechanism may prompt the cells to undergo malignant transformation, or in other words to exhibit cancer-promoting effects.

Apoptosis occurs via two distinct pathways, namely intrinsic and extrinsic pathways. The extrinsic (death receptor) pathway occurs when the death receptors on the surfaces of the cells are stimulated by extracellular ligands such as TNF (tumor necrosis factor), Fas-L (Fas ligand) and TRAIL (TNF-related apoptosis-inducing ligand). The binding of the ligand to the respective receptors induces the assembly of death-inducing signalling complex or DISC, which is comprised of three components: Fas-associated death domain (FADD) and protein caspases 8 and 10. Downstream caspases such as caspase 3 and caspase 7 are then activated, thereby triggering apoptosis.

Regarding the intrinsic or mitochondrial-mediated apoptotic pathway, activation is triggered by various intra- or extracellular stress signals, such as oxidative stress, irradiation, toxic agents and others. Incoming stress signalling activates pro-apoptotic proteins such as BAX and BAK, which induces mitochondrial outer membrane permeabilization (MOMP). This action is counteracted by antiapoptotic BCL-2 family proteins (BCL-2 or BCL-X_L_) or MCL-1. Cytochrome c and SMAC are then released from the mitochondrial intermembrane space into the cytosol. Cytochrome c binds with apoptotic protease-activating factor 1 (APAF1) to form apoptosome, which functions by activating caspase 9 and goes on to further activate caspase 3 and caspase 7, ultimately leading to apoptosis. SMAC aids in the apoptotic pathway by inhibiting the caspase inhibitor X-linked inhibitor of apoptosis protein (XIAP).

**Figure 5:**
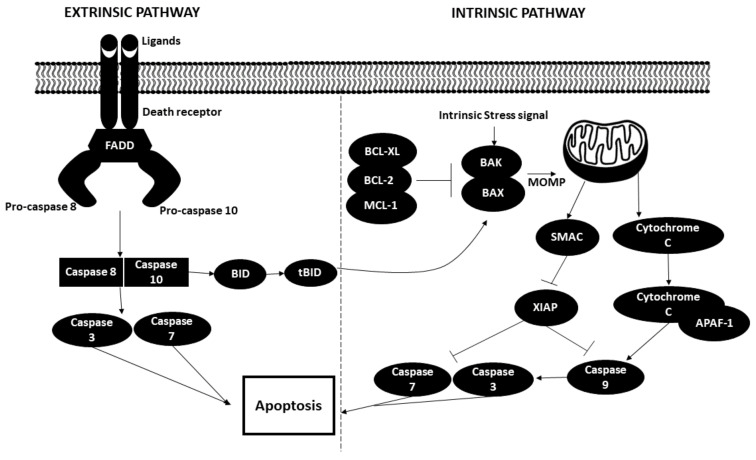
Extrinsic and intrinsic pathways in apoptosis. BID: BH3-interacting domain death agonist; BCL-xL: B cell lymphoma extra-large; BCL-2: B cell lymphoma 2; MCL-1: myeloid cell leukemia sequence 1; BAK: Bcl-2 homologous antagonist killer; BAX: Bcl-2-associated X protein; MOMP: mitochondrial outer membrane permeabilization; SMAC: Second mitochondrial derived activator of caspases; XIAP: X-linked inhibitor of apoptosis protein; APAF-1: Apoptotic protease activating factor 1.

Both extrinsic and intrinsic pathways merge via the action of caspase 8, which cleaves and activates BH3-interacting death domain agonist (BID). BID exerts its role by activating BAX and BAK [75,76,77]. The apoptotic process is illustrated in Figure 5.

#### The Role of miRNAs in Altering the Apoptotic State of Cancer Cells

The miRNAs can regulate apoptosis through promotion or inhibition, depending on the target. MiR-224 is overexpressed in breast cancer and directly targets CASP9 (caspase 9), thereby inhibiting apoptosis [78]. A recent finding reported that XIAP, an antiapoptotic gene, is negatively regulated by a novel miRNA, miR-CHA1, leading to apoptosis induction in non-small cell lung cancer [79]. APAF1, an activator of caspase 9 in the intrinsic pathway, is inhibited by miR-484 in non-small cell lung cancer, thereby abrogating apoptosis [80]. Other examples of miRNAs involved in apoptosis are depicted in Table 4.

### 3.4. Senescence

Senescence refers to irreversible cell cycle arrest. Senescence can be divided into three different groups: replicative, stress-induced and oncogene-induced senescence. Replicative senescence occurs due to telomere shortening, which is largely responsible for cell replication. Stress-induced senescence occurs in response to various stress stimuli, such as radiation, oxidative stress, cytotoxic agents and other genotoxic stress. Oncogene-induced senescence takes place following overexpression of oncogenes such as RAS^G12V^ or BRAF^V600E^ [91]. Senescence can act as a potent barrier against carcinogenesis by halting cell proliferation and forcing the cell to undergo permanent cell cycle arrest. Dysregulation of the senescence pathway may either promote or inhibit malignant transformation [92].

**Figure 6:**
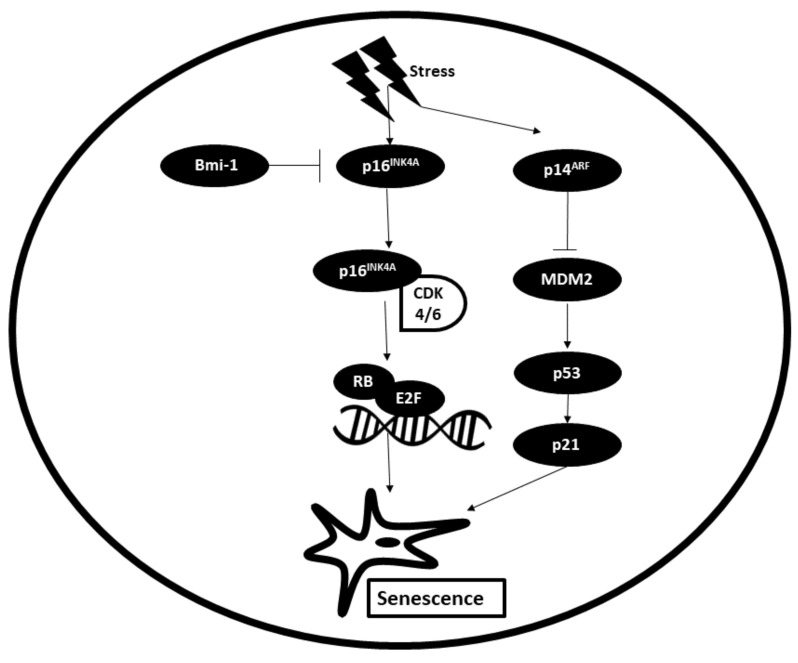
The main regulators of the senescence pathway. Bmi-1: B-cell-specific Moloney murine leukemia virus integration site 1; RB: Retinoblastoma; CDK: Cyclin-dependent kinase; MDM2: Mouse double minute 2 homologue.

The two main signalling pathways involved in senescence are the p16/pRB pathway and p53/p21 pathway. Upon receiving stress signalling from upstream regulators, p16^INK4A^ (cyclin-dependent kinase inhibitor) is activated. Bmi-1 (B-cell-specific Moloney murine leukemia virus integration site 1) acts as a negative regulator of p16. Activation of p16 results in its binding to CDK4/6, thereby inhibiting the phosphorylation of pRB (retinoblastoma protein). Inactive Rb blocks cell cycle progression by binding to and inactivating transcription factor E2F, which promotes the entry of cells from G1 to S phases. With this process, cell cycle progression is blocked and senescence is induced. Upstream signalling upregulates the expression of p14^ARF^, which functions by inhibiting the activity of MDM2, a p53 inhibitor. Following this, p53 is activated, which in turn induces the expression of its downstream effector p21, a cell-dependent kinase inhibitor (CKI) that blocks cell cycle progression by inhibiting the formation of the cyclin–CDK complex. This in turn leads to activation of senescence [93]. The process of senescence is illustrated in Figure 6.

#### The Role of miRNAs in Altering the Senescent State of Cancer Cells

The senescence pathway is highly regulated by miRNA and can cause positive or negative effects on senescence, depending on the target genes affected. For example, Bmi-1, a negative regulator of p53, is downregulated by miR-128 overexpression, thereby directly inducing senescence in glioma cells [94]. Furthermore, miR-30 evades senescence by indirectly targeting two key senescent effectors, p16 and p53, via the downregulation of CHD7 and TNRC6A, respectively. CHD7 is a cotranscriptional activator of p16, whereas TNRC6A is involved in p53 activation [95]. Other examples of miRNAs involved in senescence are listed in the Table 5.

## 4. Hallmark 3: Vascularization

Vascularization is another hallmark of cancer, whereby cancer cells promote the formation of blood vessels to deliver nutrients for fast-growing solid tumors. The most well-known process of vascularization is angiogenesis. In normal cells and tissues, the angiogenesis is a controlled process that is turned on or off depending on the needs of the cells; however, in cancerous cells and tumors, the angiogenesis process is continuous and there is a dysregulation of pro- and antiangiogenesis factors [104]. This continuous activation of angiogenesis allows the cancer cells to form blood vessels to obtain sufficient nutrients for continuous growth and proliferation. There are other ways tumors can achieve vascularization, such as vascular co-option, intussusceptive microvascular growth and vasculogenic mimicry [105].

### 4.1. Vascularization Mechanisms in Cancer Cells

Vascularization, also known as angiogenesis, is the formation of new blood vessels surrounding a solid tumor into other ducts within the body. Vascularization generally starts when a solid tumor grows to a certain size, as this creates the need for extra nutrients and oxygen to be supplied to the tumor microenvironment for propagation of the primary tumor. This is triggered when there is low oxygen within the tumor microenvironment (Hypoxia). Hypoxia induces HIF1-α (hypoxia-inducible factor-1 alpha) expression, leading to the activation of downstream factors that are crucial for vascularization [106,107,108].

VEGF (vascular endothelial growth factor) is an HIF1-α induction-dependent factor and a potent inducer of tumor vascularization. It was found that anthracycline treatment in prostate cancer-xenografted mice, which blocks the HIF1-α DNA binding potential, attenuates vascular formation via downregulation of the VEGF activity. The results also showed that the reduction of VEGF leads to impaired growth of prostate cancer [109].

In addition, cellular protease was also found to be a contributor in tissue vascularization. An example is matrix metalloproteinase (MMP), a protease that is transcriptionally activated by HIF1-α [110,111]. It was found that fibroblasts surrounding the tumor could also affect angiogenesis; fibroblasts secrete factors crucial for MMP production in neighboring tumor cells [112]. Furthermore, downregulation of MMP attenuates angiogenesis, further supporting the suggestion that vascularization is MMP-dependent [113].

The changes in the genes mentioned earlier affect angiogenesis by modulating the tumor microenvironment, thereby affecting crucial proteins found most predominantly in tight junctions, as well as other cell-to-cell junctions, such as adherens junctions and desmosomes. Additionally, exosomal secretion into the extracellular matrix (ECM) could also affect cell-to-cell junctions, which contribute to angiogenesis [114,115].

**Figure 7:**
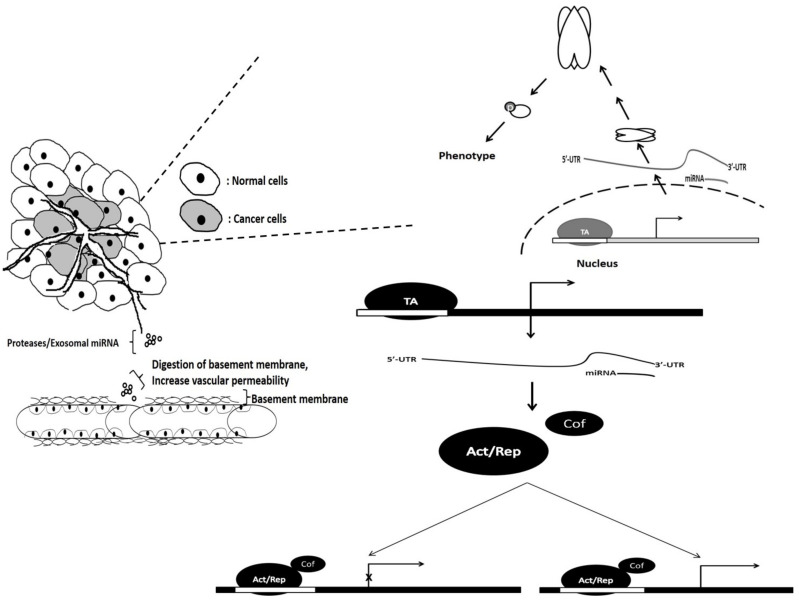
The microRNA-regulation-targeting genes involved in cancer angiogenesis, which occurs at the proteomic, genomic, exosomic and phenotypic levels. Act/Rep: Activator/repressor, Cof: Cofactor, miRNA: micro-RNA, UTR: untranslated region, TA: transcription activator, P: phosphate group.

The regulation of vascularization via miRNA can be either direct or indirect. Direct regulation can be observed when the miRNA targets both activator and suppressor genes involved in tissue vascularization via 3′-UTR binding on their mRNAs. Similar miRNA–mRNA hybrids occur through indirect regulation; however, these miRNAs target specific factors (transcription cofactors) that influence genes involved directly in vascularization. Control can occur at different levels (exosomal, proteomic, genomic and transcript) of the central dogma of molecular biology, thereby leading to angiogenesis. The microRNA regulation associated with cancer angiogenesis is illustrated in Figure 7.

### 4.2. The Role of miRNAs in the Vascularization of Cancer Cells

Cancer tissue vascularization requires specific signalling from various factors for its formation. These factors are regulated by miRNAs. Two high-risk miRNAs, namely miR-148a and miR-30, which regulate HIF1-α via binding directly to its inhibitor FIH1 (factors inhibiting HIF1-α) in the glioblastoma was reported by Wong et al. [116]. Inhibition of these miRNAs results in the downregulation of the HIF1-α protein, which corresponds to the reduction of VEGF expression and attenuation of vascularization. This is an example of the effects of cofactor targeting via miRNA binding, which influences the activity of transcription factors that directly activate gene expression.

Another interesting miRNA control process occurs when the cancer itself secretes miRNA via exosomes, thereby affecting neighbouring cells. In this case, these would be endothelial cells, which allow for high vascular permeability. This was observed in colorectal cancer cells (CC), whereby exosomal secretion from the CCs containing the miR-25-3p significantly affected the vascular integrity [117]. Another study also found that hepatocellular carcinoma cells (HCCs) overexpressed miR-210, which was found in high abundance in HCC secretion (HSS). Further experimentation revealed exosome-rich miRNA, whereby treatment of HSS on HepG2 resulted in the induction of tubal formation by downregulating SMAD4 and STAT6. Furthermore, direct targeting of the miRNA processing via DROSHA downregulation attenuates angiogenesis [118]. Other examples of miRNAs involved in vascularization are shown in Table 6.

## 5. Hallmark 4: Invasion and Metastasis

The process of vascularization also leads to the next hallmark of cancer, which is invasion and metastasis. This hallmark is a defining feature of malignant tumors characterized by their ability to spread and invade neighbouring tissues. Metastasis is also the main cause of cancer mortality, as the cancer cells travel from the tissue of origin and colonize other distant organs or tissues. This leads to cancerous growth on multiple sites in the body, which compromises the bodily function and ultimately leads to death [142].

### 5.1. Mechanisms of Invasion and Metastasis in Cancer Cells

**Figure 8:**
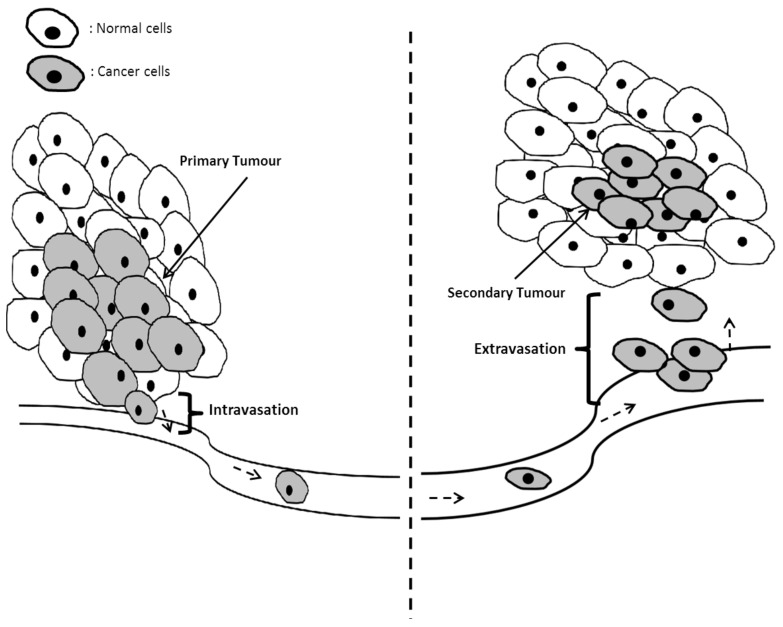
A brief overview of cancer metastasis. Cancer cells loses their adhering factors, leading to intravasation, a process whereby the cancer cells detach from the primary tumor site and invade the circulatory system. Once they are in the circulatory system, cancer cells will be disseminated throughout the body, followed by extravasation, where the cancer escapes the circulatory system and forms a secondary tumor.

Proliferation of cancer cells can be continuous post-vascularization, as there are now means for providing nutrients to the primary tumor. Metastasis usually follows vascularization. Metastasis is a process of invasion where the primary tumor obtains the means to propagate to other parts of the affected individuals from the primary tumor site. A brief overview regarding metastasis is illustrated in Figure 8.

This occurs due to the loss of adhering factors found within the cell-to-cell junctions, which are crucial for intercellular attachment [143,144,145,146]. The junctions affected are desmosomes, adherens junctions, tight junctions and gap junctions [147,148,149].

Proteins (adhering factors—AF) found on these sites are crucial in intercellular crosslinking (desmosomes), signal exchange (tight junction or gap junction) and cytoskeletal connection (Adherent Junction). Aberrant changes of the AFs can affect cell-to-cell adhesion, allowing the tumor to metastasize [150,151]. Some of the examples of adhering factors found on cell-to-cell junctions and their implications in metastasis are shown in Table 7.

### 5.2. The Role of miRNAs in Metastases and Invasion of Cancer Cells

Dysregulation of gene expression usually leads to cancer metastasis. Some cancer cells acquire the characteristics of other cell types in order for them to metastasize. An example can be seen in breast cancer bone invasion, whereby miR-301a-d regulates DKK-1, RUNX-2 and ITGA5 genes, which are involved in osteogenesis, leading to breast cancer osteomimicry. Low expression of miR-30 was observed in tumor samples, and induction of miR-30 expression was followed by attenuation of bone metastases [162].

Furthermore, mir-331 and miR-195 were also reported to have metastatic implications. It was found that these circulating miRNAs could be prognostic markers in luminal A breast cancer. Furthermore, mir-331 and miR-195 target a cohort of genes that is crucial for Akt signalling and epithelial mesenchymal transition (EMT), in which both are crucial for metastasis [163]. Various other examples of miRNAs implicated in cancer invasion and metastasis are shown in Table 8.

## 6. Metabolic

### 6.1. Drivers of Metabolic Reprogramming

**Oncogene RAS:** Oncogenic RAS is frequently upregulated in cancer and its aberrant signalling contributes to altered metabolism. RAS signalling promotes glucose uptake by upregulating the expression of glucose transporter GLUT1. RAS signalling also stimulates the glycolytic pathway, which is the master regulator of aerobic glycolysis, also known as the Warburg effect [178].

**Oncogene MYC:** MYC functions as a transcription factor and is involved in various oncogenic processes. Aberrant MYC signalling is common in cancer, leading to altered metabolism. Active MYC signalling is associated with upregulation of metabolic enzymes, such as lactate dehydrogenase A (LDHA) and pyruvate kinase (PKM2) of the glycolytic pathway [179,180]. MYC also induces the utilization of glutamine as an alternative energy source [181].

**Tumor suppressor 53 (TP53):** Loss of p53 can contribute to alterations in metabolic pathways. Furthermore, p53 impairs glucose metabolism by inhibiting the transcription of glucose transporters GLUT1 and GLUT4 [182]. The glycolysis pathway that is preferentially utilized by cancer cells is impaired by p53 through direct downregulation of hexokinase 2, an enzyme involved in glycolysis and indirectly through inducing the expression of PARK2, a negative regulator of HIF-1a [183,184].

**PI3K-Akt-mTOR and AMPK signalling:** Hyperactivation of PI3K-AKT-mTOR signalling is frequently observed in various cancers. PI3K-AKT-mTOR positively regulates glucose uptake and glycolysis in cancer by exerting its action on glucose transporter 1 (GLUT1) [185]. Activation of GLUT1 results in the upregulation of its downstream target, HIF-1a. AMPK signalling indirectly induces GLUT1 activity by inhibiting the negative regulator of GLUT1, TXNIP [186].

**Hypoxia-inducible factor 1 (HIF-1):** HIF1 is a transcription factor that is stimulated in response to hypoxia. Since the hypoxic environment is commonly found in most cancers, there is no doubt that HIF1 is also frequently upregulated in cancer cells. Additionally, HIF1 signalling can be activated by other factors such as oncogenes. One of the main roles of HIF is as a master regulator of aerobic glycolysis or the Warburg effect in cancer cells. HIF1a activates the glycolytic pathway by upregulating glucose metabolism enzymes, such as LDHA, PKM2, HK1 and HK2, and by increasing glucose transporters (GLUTs) [187,188,189]. HIFs are negatively regulated by von Hippel–Lindau protein (pVHL) and factor-inhibiting HIF1 (FIH-1) [190,191].

### 6.2. Alteration of Metabolic Pathways in Cancer Cells

**Figure 9:**
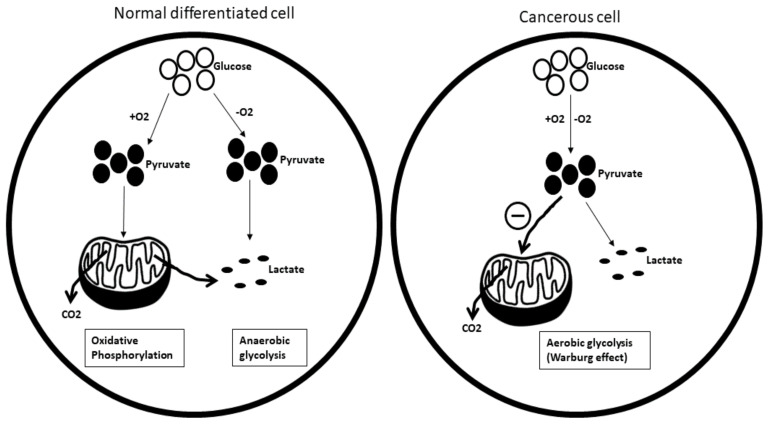
The differences in glucose metabolism between normal differentiated cells and cancerous cells. O_2_: oxygen; CO_2_: carbon dioxide.

**Carbohydrate metabolism:** One of the key players associated with cancer metabolism is glucose. Cancer cells require a greater abundance of glucose than normal differentiated cells to meet their higher energy demand. Instead of relying on more efficient oxidative phosphorylation for glucose production, cancer cells choose to opt for less efficient glycolysis, even under nor-moxic conditions. This phenomenon is referred to as the ‘Warburg effect’ or as aerobic glycolysis (Figure 9).

Glycolysis supplies the cancer cells with the building blocks that are needed for macromolecule synthesis [192,193]. Cancer cells reprogram glucose metabolism to work in their favor through various mechanisms, with a few examples being increasing the number of glucose transporters (GLUTs) and upregulating glycolytic enzymes such as hexokinase, pyruvate kinase and lactate dehydrogenase [194]. The end product of glycolysis, pyruvate, then feeds into the tricarboxylic acid cycle (TCA) to produce citrate as an alternative energy source.

**Lipid metabolism:** Lipid metabolism is often deregulated in cancer and mostly contributes positively to cancer development. Lipids are of great importance for cells, as they are needed to form the lipid bilayer of plasma membrane, which aids in cell proliferation. De novo lipid biosynthesis by cancer cells exhibits greater resistance against oxidative stress. Furthermore, lipid metabolism contributes to carcinogenesis by providing alternative energy sources and in the synthesis of signalling molecules, such as hormones. Examples of crucial genes involved in lipid metabolism are sterol regulatory element-binding protein (SREBP), fatty-acid-binding protein (FABP) and adipose-differentiation-related protein (ADRP), while the crucial enzymes are fatty acid synthase acetyl-coA synthetase (ACSL), acetyl-CoA carboxylase (ACC) and others [195,196].

**Amino acid metabolism:** Amino acids are needed as the building blocks for protein synthesis. Glutamine, a critical amino acid, is utilized by cancer cells to produce glutamate for use in the TCA cycle or for glutathione synthesis in the antioxidant system. The main enzyme that participates in this pathway is glutaminase (GLS) [197,198].

### 6.3. The Role of miRNAs in Metabolic Rewiring

The miRNAs participate in the regulation of metabolic rewiring by either inducing or inhibiting the expression of metabolic-related genes. For example, GLUT1 and HKII are negatively regulated by miR-124, which subsequently leads to impaired glycolysis in non-small cell lung cancer [199]. A recent report demonstrated that miR-31-5p can promote the Warburg effect by downregulating HIF-1a inhibitor (FIH) activity, which ultimately results in increased glycolysis and ATP production, further sustaining lung cancer cells [200]. Other examples of miRNA involved in metabolic rewiring are depicted in Table 9.

## 7. Hallmark: Tumor Microenvironment

The tumor microenvironment (TME) plays a vital role in the development, progression and eventual metastasis of cancer. It has been identified that the fundamental mechanisms governing interactions between various components of the TME and tumor cells encompass vastly dynamic factors, including hypoxia, as well as multiple cell types, such as cancer-associated fibroblasts (CAFs) and macrophages [211]. While cancer cells have been known to secrete a multitude of microRNAs to neighbouring and distant cells via exosomes to augment their functions, mounting evidence has also implicated the role of the TME in contributing to further supplementing malignant cells with factors that favor their survival and progression [211]. A recent review in this area extensively covered the influence of microRNAs in hypoxia, angiogenesis and the interplay of various cell types [211,212]. The most recent research in this area further unraveled the novel microRNAs involved in the TME, which will be covered in more detail in this section.

### 7.1. miRNAs Involved in Cancer-Associated Fibroblasts (CAFs)

The tumor microenvironment consists of various cell types. Among those that play the most vital roles in the progression of the disease are cancer-associated fibroblasts (CAFs). CAFs have been known to interact with tumors via a multitude of mechanisms, including exosomes, which convey biological instructions through the transport of metabolites, long non-coding RNA (lncRNAs), proteins and microRNAs [213]. In a reciprocal manner, exosomes from tumors are able to transform the function of CAFs, which often leads to an enhanced microenvironment favoring the survival and development of tumors, while exosomes from CAFs can be internalized by tumor cells, and in most cases can partake in the progression and metastatic formation of cancers [213].

Recent research on CAF-secreted microRNAs affecting cancers of the oral cavity revealed that they regulate tumor-inhibitory and tumor-promoting pathways. The study is done by using oral squamous cell carcinoma (OSCC) patient samples identified that miR-382-5p was overexpressed in CAFs as compared to normal fibroblasts. In vitro assays showed that CAFs overexpressing miR-382-5p promoted the migration capabilities and invasiveness of OSCC cells [214]. In contrast, another research demonstrated that miR-34a-5p was able to repress OSCC cell proliferation and metastasis. This microRNA, which directly targets AXL, was also found to be capable of inhibiting tumorigenesis in xenograft models. Collectively, this study indicated that activation of the miR-34a-5p/AXL axis was able to confer aggressiveness to OSCC via the AKT/GSK-3β/β-catenin/Snail signalling cascade [215].

A recent study revealed that exosomal miR-139 from CAFs was able to repress the progression of gastric cancer by inhibiting matrix metalloproteinase 11 (MMP11) [216]. Initial experiments indicated that there is a significant downregulation of miR-139 in CAFs of the gastric cancer microenvironment. Further analysis indicated that MMP11 was the direct target of miR-139. The researchers attempted to shuttle miR-139 into CAFs to increase their bioavailability to gastric cancer cell lines in vitro and to stomach tumors in vivo, and found that both experiments resulted in drastic decreases in MMP11 expression. Further analysis showed significant reductions in invasiveness in vitro and repression of tumor progression and metastasis in vivo. The data from this study indicated that miR-139 produced in gastric CAFs may repress the progression and development of gastric cancer metastasis by modulating the level of MMP11 in the surrounding tumor microenvironment. Another study showed that the suppression of miR-214 in CAFs leads to increased migration and invasion abilities of stomach cancer cells [217]. This study further revealed that these characteristics were induced by the microRNAs’ target FGF9, which is linked to the further development of EMT. Experimentation regarding the overexpression of this miRNA suppressed the migration and invasion of gastric cancer cells in vitro. Moreover, it was found that the use of mimetics led to elevation of E-cadherin and suppression of Vimentin, N-cadherin and Snail, denoting repression of EMT of GC cells; thus, this study indicates that miR-214 is able to repress the tumor-promoting capabilities of CAFs via targeting of FGF9 to regulate the EMT process of gastric cancer cells.

Interestingly, some microRNAs have been found to suppress the conversion of normal fibroblasts into cancer-associated fibroblasts. It was demonstrated that miR-124 produced by human ovarian surface epithelial cells (hOSECs) was able to suppress the conversion of normal fibroblasts into cancer-associated fibroblasts (CAFs) in ovarian cancer [218].

Analysis of exosomal miRNA of ovarian cancer samples showed a marked decrease in the expression of miR-124, whereas the opposing finding was found in normal hOSECs. Further analyses indicated that normal fibroblasts with suppressed miR-124 exhibited characteristics of CAFs, including upregulation of α-SMA and FAP, which led to enhanced migratory and invasive capabilities. Experiments to reverse the condition via ectopic expression of miR-124 in CAFs led to the attenuation of α-SMA and FAP expression and counteracted motility and invasion traits. Further, the direct target for miR-124 is the sphingosine kinase 1 (SPHK1) gene transcript involved in the regulation of cell proliferation, adhesion, chemotaxis, migration and tumor growth, among others. This study provided evidence that ovarian cancers, via downregulation of miR-124, mediate the CAF transition to mold the tumor microenvironment for optimal oncogenesis. Further to this, it was reported that miR-141-3p was able to attenuate gastric-cancer-mediated transformation of normal fibroblasts and BMSC into CAFs. Normal fibroblasts with suppressed miR-141-3p exhibited features of CAFs, including enhanced migratory and invasive capabilities. Additionally, miR-141-3p was able to hinder the migration and invasion of gastric cancer cells and repressed the transformation of normal fibroblasts and BMSC into CAFs. Additionally, it was identified that the direct target for miR-141-3p is the STAT4 gene transcript. These data collectively demonstrated that miR-141-3p exerts its actions by regulating the STAT4/wnt/β-catenin pathway [219].

### 7.2. miRNAs in Hypoxia

Hypoxia occurs naturally in the tumor microenvironment (TME) as a result of oxygen deprivation due to cancer growth, and is able to alter cell-to-cell interactions, as well as molecular signalling. The roles of microRNAs in the hypoxic TME have previously been shown elsewhere [211,212].Since then, several research groups have identified additional miRNAs that regulate key pathways during hypoxia, which will be covered in the following section.

Numerous research publications have linked the onset of hypoxia to the acquisition of increased cellular proliferation, migration and invasiveness of cancer cells. It is noteworthy that although hypoxic tumors tend to activate these same capabilities but often activate different mechanisms, different pathways and microRNAs are employed to achieve this end. A recent study demonstrated that miR-590-5p is induced under low-oxygen conditions in colorectal cancers and is able to aid in disease progression by regulating the activity of matrix metalloproteinases. RECK, the direct target of this miRNA, enhances the invasive and migratory ability of cancer cells when suppressed by activating matrix metalloproteinases (MMPs) and filopodia protrusions; thus, this study showed that downregulation of miR-590-5p leads to inhibition of tumor proliferation and metastasis in mouse models of CRC [220].

Further, recent research on hepatocellular carcinoma (HCC) identified that miR-196-5p is inducible under hypoxic conditions and contributes to tumor progression and liver cancer metastasis. Clinical samples from HCC patients showed significantly low levels of miR-196-5p, with further in vitro and in vivo experiments on the ectopic overexpression of this miRNA, demonstrating substantial impairment of HCC growth and metastasis. Additionally, it was found that miR-196-5p exerted its function through regulation of the high-mobility group AT-hook 2 (HMGA2) gene transcript [221].

In another related study, it was discovered that miR-210 mediated the epithelial–mesenchymal transition (EMT) in pancreatic cancers under hypoxic conditions [222]. It was reported that as the level of miR-210 increased under hypoxia in PANC-1 cell lines, the expression of HIF-1α and NFκB also elevated in tandem whilst HOXA9 decreased. HOXA9 was proven to be the direct target of miR-210. Ectopic expression of miR-210 under normoxic conditions led to decreasing levels of EMT epithelial markers, which included E-cadherin and β-catenin, while increasing the expression of mesenchymal markers, including vimentin and N-cadherin. This led to the net result of increasing cell migration and invasiveness. Additionally, it was disclosed that NFκB levels also increased, further enhancing the migration and invasiveness of the cells. In contrast, experiments using miR-210 antagonists on hypoxic PANC-1 cells showed reversal of the EMT, HOXA9 and NFκB gene expression, which led to decreased cell migration and invasiveness. The data from this study collectively showed that under hypoxic conditions, miR-210 suppressed levels of HOXA9 to activate the NFκB pathway, which drives EMT in pancreatic cancer.

Studies on specific microRNAs found in different types of cancer cells often show that they target different mRNA transcripts to exert their function, thereby affecting dissimilar pathways. This has been observed with miR-210, which was found to target HOXA9 in pancreatic cancers, although for prostate cancers that target was determined to be the neural cell adhesion molecule (NCAM). A recent study identified that miR-210 expression in prostate cancer is induced by hypoxia and is involved in regulating neural cell adhesion [223]. Taken together, these studies show that miR-210 plays a vital role in regulating cellular responses to hypoxia and provides evidence that the regulation of various adhesion molecules is crucial for the progression of multiple cancers.

In addition to cell migration and adhesion molecules, it has been observed that hypoxia of the TME also induces the dysregulation of microRNAs affecting cellular metabolism. In a recent study on liver cancer, it was discovered that miR-885-5p directly targets Hexokinase 2 (HK2) to regulate the Warburg effect [210]. This study revealed marked suppression of miR-885-5p in HCC tissues and cell lines. Ectopic expression of miR-885-5p in hypoxic models of HCC led to substantial suppression of growth and migration in in vitro and in vivo models. Further, the overexpression of miR-885-5p in vitro led to marked reductions in glucose uptake and lactate production via suppression of several glycolytic enzymes, thereby providing evidence that it is involved in the regulation of the cancer cells’ glycometabolic activity. Additional analysis also revealed that miR-885-5p binds to the 3′ UTR transcript of hexokinase 2. The data from this study collectively suggested that the miR-885-5p/HK2 axis has additional potential to be explored as a therapeutic target and prognostic biomarker of liver cancer. A further summary of the microRNAs that control the tumor microenvironment is shown in Table 10.

## 8. Hallmark 7: Immune Modulation

Cancer cells are constantly being monitored and eliminated by the immune system; therefore, cancer cells must have a way to avoid detection by the immune system, which is achieved via immune modulation. This achieved is through cancer immunoediting, which involves the selection of cancer cells that are able to evade the immune system. Cancer immunoediting involves three phases, namely elimination, followed by equilibrium and finally escape [233]. The role of miRNAs in immunomodulation of the cancer microenvironment is well recognized. Recent reviews in this area have extensively covered the influence of miRNAs in immune-modulatory molecules and immune cells as well as immune checkpoints such as PD-1/PD-L1 [234,235,236,237]. The latest research in this area has further unraveled the novel miRNAs that contribute to the survival of tumors by dysregulating several key elements of the immune system involved in the surveillance of cancers, including tumor-associated macrophages (TAMs), T cells, myeloid-derived suppressor cells (MDSC) and natural killer (NK) cells, which will be covered in more detail in this section.

### 8.1. miRNAs and Tumor-Associated Macrophages

The roles of tumor-associated macrophages (TAMs) in cancers have been well studied. TAMs are known to exhibit functional plasticity, which enables wide-ranging phenotypes from immune-stimulating to immune-suppressive states [238]. The phenomenon of TAM reprogramming and expansion to promote oncogenicity is purportedly influenced by tumor-derived factors [239].

Cancers that carry the p53 mutation have reportedly been able to reprogram macrophages to support tumor growth via exosomes containing miR-1246. Colon cancer cells expressing mutp53 release miR-1246-enriched exosomes to adjacent macrophages, which reprograms them into an anti-inflammatory immunosuppressive state, favoring enhancement of TGF-β activity [240]. This immunomodulatory mechanism alters the microenvironment substantially to induce cancer progression and subsequent metastasis, leading to poor survival of colon cancer patients.

Recent research has also showed that high expression of miR-21 in TAMs is associated with the progression of tumor growth [239]. It was further revealed that miR-21-depleted TAMs promote the antitumor response by undergoing transcriptional network rewiring, which contributes to a proinflammatory angiostatic state. This results in enhancement of cytotoxic T cell activity via expression of cytokines and chemokines, showing that regulation of miR-21 has potential therapeutic implications.

Aside from tumors, transformed TAMs are also able to secrete miRNA-enriched exosomes to further regulate the microenvironment to favor cancer progression. It was identified in epithelial ovarian cancer (EOC) samples that miR-29a-3p and miR-21-5p released by TAMs to neighbouring CD4^+^ T cells were able to inhibit STAT3 expression. Additionally, these miRNAs also induce imbalance of Treg/Th17 cell regulation, leading to further inhibition of STAT3 [241]. These data imply the possible development of an EOC treatment by targeting exosomal miRNAs.

Recently, research has shown that miR-148b, which controls the expression of colony-stimulating factor 1 (CSF1), is downregulated in metastatic hepatocellular carcinoma cells (HCC) [242]. The authors also reported that the dysregulation of miR-148b led to progressive growth and metastasis of HCC via CSF1/CSF1 receptor-mediated TAMs infiltration, concluding that miR-148b plays a suppressor role in HCC.

### 8.2. miRNAs and Myeloid-Derived Suppressor Cells (MDSCs)

Myeloid-derived suppressor cells (MDSCs) are a subset of cells normally present in infection and cancer microenvironments. This heterogeneous group of cells are known to have immunosuppressive activities, although their exact role has not been elucidated.

It was recently discovered that MDSC expansion in gastric cancers was induced by miR-107 secreted by tumor cells [243]. The authors found that miR-107 was not only abundant in gastric cancer cells but also in the secreted exosomes. The exosomes, when taken up by MDSCs, target and suppress expression of DICER1 and PTEN genes, which are responsible for regulating MDSC proliferation and activation of the PI3K pathway, respectively. This study concluded that gastric cancers were able to induce the expansion and activation of MDSC, while the downregulation of miR-107 serves as a novel therapeutic intervention for gastric cancer.

Another study showed that a subset of MDSCs of the stomach express miR-130b during helicobacter-induced spasmolytic polypeptide-expressing metaplasia (SPEM) [244]. It was determined that mir-130b was required for T cell proliferation suppression and that its levels in the blood correlate to the metaplastic changes of the stomach. These data imply that expression of miR-130b in gastric MDSCs could be explored as a marker for metaplastic changes that potentially lead to stomach cancer.

The presence of MDSCs is a known impediment that negatively affects cancer immunotherapy. Huber and colleagues in their study on melanomas, determined that a set of microRNAs, which included miR-99b, miR-100, miR-125a, miR-125b, miR-146a, miR-146b, miR-155 and let-7e, is associated with MDSCs and enables treatment resistance via immune checkpoint inhibitors. These microRNAs, which have been shown to be prevalent in tumor samples, CD14^+^ monocytes and plasma, were determined to be responsible for converting monocytes into MDSCs and were correlated with myeloid cell infiltration. The abovementioned MDSC-related microRNAs were, therefore, indicated as plausible blood markers for the prediction of immunotherapy outcomes [245].

In a recent study on glioma cells, it was shown that miR-10a and miR-21 found in glioma-derived exosomes (GDE) were responsible for initiating an MDSC-induced immunosuppressive microenvironment [246]. In their study, it was demonstrated that GDE effects on MDSCs were achieved through targeting of RAR-related orphan receptor alpha (RORA) and phosphatase and tensin homolog (PTEN) pathways. This study concluded that glioma cells are able to exert extensive differentiation and activation effects on MDSCs through secreted exosomes under hypoxic conditions.

### 8.3. miRNAs and Natural Killer (NK) Cells

Natural killer (NK) cells have been identified as leading effector lymphocytes of the innate immune system against the formation of tumors. Currently, it is widely accepted that the cytotoxic activity of NK cells is decreased in many forms of cancers. These observations have been postulated to be related to microRNA dysregulation.

In a recent study, it was found that miR-20a elevation in colorectal cancer (CRC) cells was responsible for evasion of immune surveillance by NK cells [247]. Although preliminary investigations on this miRNA indicated that overexpression and knockdown did not affect CRC cell growth in vitro, further cytotoxicity assays showed that miR-20a knockdown increased CRC cell sensitivity to NK cell activity. The direct target of miR-20a was identified to be NKG2D ligand major histocompatibility complex (MHC) class-I-related chain gene A (MICA) transcripts. This study, therefore, postulated that miR-20a targets MICA to regulate the sensitivity of CRC cells to NK cells.

In another recent study, it was demonstrated that miR-130a targets STAT3 to increase the cytotoxic activity of NK cells against non-small cell lung cancer (NSCLC) cells [248]. The findings indicated that miR-130a was markedly reduced and STAT3 was notably elevated in NK cells isolated from NSCLC patients. Further functional studies by overexpressing miR-130a reversed the capability of NK cells to increase cytotoxicity against A549 lung cancer cells. In a related study on lung cancer, miR-218-5p was able to suppress the cytotoxic activity of NK cells towards lung adenocarcinoma (LA) by targeting serine hydroxymethyl transferase 1 (SHMT1). Further experimentation by attenuating miR-218-5p resulted in IFN-γ and TNF-α secretion in IL-2-activated NK cells [249]. These studies demonstrated that manipulation of microRNAs is a viable strategy for potentiating NK cell immunotherapy against multiple types of lung cancers.

It was additionally discovered that the microRNA cluster Mirc11 was able to disrupt inflammatory responses of NK cells but not their cytotoxic activity against B16-F10 melanoma. The loss of the Mirc11 cluster, which consists of miRNA-23a, miRNA-24a and miRNA-27a, appears to significantly reduce the expression of proinflammatory factors in in vitro experiments and also hindered interferon-γ-mediated clearance of melanoma in animal models by NK cells [250].

In a recent study on liver cancers, it was demonstrated that HCC metastasis in the lungs was driven by miR-561-5p/CX3CL1 signalling [251]. This study disclosed that three miRNAs, namely miR-137, miR-149-5p and miR-561-5p, were identified to be present in patients with pulmonary metastasis stemming from HCC. Bioinformatics analyses and chemokine expression profiling determined CX_3_CL1 to be the probable target of miR-561-5p. Moreover, it was found that high levels of this miRNA were responsible for attenuating the anticancer activity of CX_3_CG1^+^ NK cells via CX_3_CL1; therefore, these results demonstrated that downregulation of miR-561-5p in CX_3_CG1^+^ NK cells could potentially be a strategy for developing cellular anticancer treatment effectors.

### 8.4. miRNAs and T Cells

T cells are major constituents of the adaptive immune system and are capable of distinguishing altered cancer cells from normal cells; however, wide-ranging immunosuppressive mechanisms found in the tumor microenvironment to evade detection enable the continuing survival of tumors, and in some cases further deteriorate prognosis through transforming the functions of T cells.

In recent research, it was revealed that miR-24-3p hinders T cell activity by targeting FGF11 in nasopharyngeal carcinoma (NPC) [252]. In this study, enrichment of miR-24-3p was observed in exosomes of NPC cell line and patient sera samples. Knockdown experiments reversed the inhibition of T cell proliferation, Th1 and Th17 differentiation and induction of Tregs. It was also discovered that miR-24-3p directly targets FGF11 for its activity, while tumor FGF11 levels were positively correlated to CD4^+^ and CD8^+^ T cell counts in vivo, which were predicative of favorable patient disease-free survival.

It is widely accepted that progressing tumors derive mechanisms to hijack the PD-1/PD-L1 immune checkpoint via microRNAs to dysregulate T cell functions [237]. MiR-140 was significantly suppressed in *Helicobacter pylori* (Hp)-positive gastric cancers [253]. PD-L1 was identified to be the direct target of miR-140 in patient samples. Further experimentation to overexpress miR-140 demonstrated that gastric cancer proliferation could be suppressed through the regulation of PD-L1 levels. Moreover, in vivo research also showed that miR-140 repressed the growth of tumors in mice models of gastric cancer. The increase in cytotoxic CD8^+^ T cells and reduction of MDSC and Tregs in the immediate tumor microenvironment were determined to be the main factors contributing to the effects of miR-140 treatment. These data collectively indicate that miR-140 was able to target PD-L1 to exert an antigastric cancer response.

In recent research on breast cancer, it was discovered that miR-149-3p plays a vital role in resuscitating CD8^+^ T cell deletion by downregulating inhibitory receptors and enhancing cytokine secretion [254]. The researchers found that PD-1-overexpressing CD8^+^ T cells showed significantly lower levels of miR-149-3p, which was predicted to bind to the 3′UTRs of T cell inhibitor receptors PD-1, TIM-3, BTLA and Foxp1 mRNA transcripts. By using mimetics of miR-149-3p to treat CD8^+^ T cells, the authors managed to enhance their killing capacity on 4TI mouse breast tumor cells, which was largely attributed to reversal of T cell inhibitor receptor expression, reduction of apoptosis and secretion of effector cytokines, including IL-2, TNF-α and IFN-γ. Based on these findings, it was speculated that miR-149-3p could potentially be developed into an effective antitumor immunotherapeutic agent.

In studies on colon cancer, the indoleamine 2,3-dioxygenase 1 (IDO1) transcript was identified as the target for miR-448 to regulate the antitumor function of CD8^+^ T cells [255]. In vivo experiments indicated that overexpression of IDO1 promoted xenograft tumor growth in immune-competent mice but not in nude mice. Additional studies on the downregulation of IDO1 via ectopic expression of miR-448 mimetics markedly reduced IDO1 protein expression levels, which unequivocally led to the inhibition of apoptosis of CD8^+^ T cells. The findings in this study suggest that miR-448 is able to suppress IDO1 to enhance CD8^+^ T cell activity against colon cancers.

In silico approaches to identify the correlations of miRNAs to cancers have also become increasingly important due to the increasing wealth of bioinformatics data. One such study identified that miR-195 is potentially involved in inhibiting lung adenocarcinoma progression by enhancing CD4^+^ T cell activation [256]. Further analysis identified that CD4^+^ T cells were the subset of lymphocytes involved in infiltration of lung adenocarcinoma through activation of miR-195-targeted genes. The data from this study collectively indicated that miR-195 is able to act as an inhibitor of lung adenocarcinoma by enhancing CD4^+^ T cell activity via the CCDC88C/Wnt signalling pathway. A further summary of miRNAs involved in immune modulation is shown in Table 11.

## 9. The Role of microRNAs in Cancer Biology beyond the Hallmarks of Cancer

Most recently, it has come to light that the hallmarks of cancer themselves are not sufficient to comprehensively describe the full length and breadth of cancer biology. A plethora of modulations that enable cancer cells to be resistant to therapy and underlying mechanisms of disease relapse have been reported and described extensively, with sufficient evidence making it now apparent that these mechanisms extend beyond the definitions of the hallmarks of cancer.

Mechanisms that have been implicated in therapy resistance and disease relapse include formation of polyploid or multinucleated giant cancer cells, which dedifferentiate somatic cells and endow them with stem-cell-like properties through the giant cell cycle, which encompasses a dormancy phase prior to reactivation and stabilization as well as the phenomenon of anastasis, in which cancer cells are able to recover themselves and become more malignant upon removal of apoptotic stimuli, senescent-like cancer cells and antiproliferative drug-resistant cancer cells [257,258,259,260,261,262]. Compounding matters further is the intratumor heterogeneity that enables different populations of cancer cells to reside within the same tumor [259]. Nevertheless, one unifying factors across all of these mechanisms is epigenetic modulation [257,258,259].

MicroRNAs have been shown to be involved in the epigenetic machineries of various types of cancer cells, which have been reviewed and described extensively elsewhere [263,264]. Some examples of microRNAs involved in epigenetic modulation include miR-200a, miR-148a, miR-19a, miR-96, miR-25 and miR-29b-3p [263,264]; therefore, it could be worthwhile further elucidating the epigenetics mechanisms that are governed by microRNAs in cancer biology, as they could provide us clues to circumvent the various hurdles currently associated with therapy resistance and disease relapse in cancer.

## 10. Conclusions

The hallmarks of cancer are the most accurate models currently available to summarize cancer biology in a nutshell. Nevertheless, as this review has shown, the hallmarks of cancer are not sufficient anymore to describe cancer biology in its entirety and could benefit from a revisit. Based on the cumulative evidence gathered herein, it would not be too far-fetched to suggest epigenetic modulation as an additional hallmark of a cancer cell. Furthermore, this review has made it clearly evident that the footprints of microRNAs are all over the various hallmarks of cancer and beyond in cancer biology. The compelling evidence demonstrates that miRNA promotes or inhibits cancer progression in various cancer types by regulating genes that could serve as either tumor suppressors or as oncogenes; thus, the outcome of miRNA regulation in the target genes could either be positive or negative in terms of cancer progression. It has been shown previously that one miRNA can regulate many genes. On that note, from this review, we can see that there is an overlap of miRNA regulation, whereby one miRNA can be involved in regulating different cancer hallmarks, which occurs by targeting many genes (Table 12). Adding to the complexity of matters is the fact that microRNAs themselves are epigenetically regulated, and in turn regulate other genes via various epigenetic modulation, creating an epigenetic feedback loop [263,264], which should be taken into consideration when studying the function and roles of microRNAs in cancer biology.

## Figures and Tables

**Table 1:** The miRNAs implicated in the selective proliferative advantage.

No	miRNA	Cancer	Target	Action	Reference
1	miR-150	Cervical cancer	FOXO4/PI3K-Akt	Downregulates FOXO4 level and promotes cell cycle progression from G1 to S phase	[21]
2	miR-132	Ovarian cancer	E2F5/Rb	Inhibits cell proliferation by targeting E2F5	[22]
3	miR-424	Endometrial cancer	E2F7/Rb	Abrogates cell cycle progression mediated by E2F7 downregulation	[23]
4	miR-133a	Hepatocellular carcinoma, osteosarcoma	IGF-1R/PI3K/Akt, MAPK/ERK	Negatively regulates IGF-1R and contributes to impaired ERK and Akt signaling pathways, which leads to reduced cell proliferation	[24,25]
5	miR-183-5p	Breast cancer	PDCD4	Downregulates the levels of p21 and p27 by targeting PDCD4	[26]
6	miR-217	Acute myeloid leukemia	KRAS/MAPK	Inhibits KRAS, which contributes to cell proliferation	[27]
7	miR-141	Nasopharyngeal carcinoma	PTEN/PI3K/AKT	Attenuation of cell proliferation occurs by suppressing PTEN, which impairs Akt activation	[28]
8	miR-136	Prostate cancer	MAP2K4/MAPK	Suppresses cell growth by inhibiting MAP2K4	[29]
9	miR-124	Breast cancer	STAT3/JAK	Impairs cell proliferation by negatively regulating STAT3	[30]
10	miR-623	Gastric cancer	Cyclin D1 (CCND1)	Renders cell cycle progression impaired by inhibiting CCND1	[31]
11	miR-129	Glioblastoma	CDK4, CDK6	Overexpression of miR-129 disrupts cell proliferation by downregulating CDK4 and CDK6	[32]
12	miR-93	Osteosarcoma	CDKN1A (P21)	miR-93 supports cell cycle progression by inhibiting p21	[33]
13	miR-196a	Laryngeal cancer	CDKN1B (P27)	Promotes cell growth by suppressing p27^Kip1^	[34]
14	miR-497	Multiple myeloma	Raf-1/MAPK	Overexpression of miR-497 suppresses cell proliferation by downregulating Raf-1	[35]
15	miR-411	Non-small cell lung cancer	SPRY4/Akt	Promotes cell proliferation by inducing Akt activation, which is suppressed by SPRY4	[36]
16	miR-101	Diffuse large B cell lymphoma (DLBL)	MAPK kinase 1 (MEK)	Abrogates cell proliferation by inhibiting MEK1	[18]
17	miR-20a	Multiple myeloma	PTEN	Negatively regulates PTEN, leading to AKT activation and cell proliferation	[19]
18	miR-590	Acute lymphoblastic leukemia (ALL)	pRB (Retinoblastoma)	Targets and downregulates Rb, leading to an increase in cell proliferation	[20]

**Table 2:** The miRNAs implicated in DNA damage.

No	miRNA	Cancer	Target	Action	Reference
1	miR-138	Small cell lung cancer	H2AX	miR-138 overexpression inhibits DNA damage repair by suppressing H2AX.	[49]
2	miR-383	Epidermoid carcinoma	ATR	Downregulates the expression of ATR, leading to defective DNA repair. Its overexpression also inhibits other DNA repair markers such as MDC-1 and GADD45	[50]
3	miR-182	Acute myelogenous leukemia	Rad51	Impairs homologous recombination repair by negatively regulating Rad51	[51]
4	miR-145	Colorectal cancer	Rad18	Negatively regulates Rad18, thereby enhancing DNA damage	[52]
5	miR-212	Glioma	BRCA1	Suppresses BRCA1, which positively regulates DNA damage repair	[53]
6	miR-205-5p	Head and neck squamous cell carcinoma	BRCA1, Rad17	Abrogates DNA repair activity by downregulating DNA repair genes BRCA1 and Rad17	[54]
7	miR-191	Osteosarcoma	Checkpoint kinase 2 (Chk2)	Inhibits Chk2, which is crucial in DDR	[55]
8	miR-142-3p	Uveal melanoma	Cdc25c	Impairs cell cycle arrest induced by Cdc25c	[56]
9	miR-33b-3p	Non-small cell lung cancer	P21	Promotes DNA damage repair by downregulating p21	[57]
10	miR-338-5p	Glioblastoma	PP2R5a	PP2R5a, a negative regulator of ATM, is inhibited, thereby promoting DNA repair	[58]
11	miR-7-5p	Small cell lung cancer Cervical cancer	PARP1	Abrogates DNA repair by downregulating PARP1	[46,47]
12	miR-203a-3p	Ovarian cancer	ATM	Promotes cell cycle arrest by inhibiting ATM	[48]

**Table 3:** The miRNAs implicated in autophagy.

No	miRNA	Cancer	Target	Action	Reference
1	miR-26	Hepatocellular carcinoma	ULK1	Abrogates autophagy initiation step by inhibiting ULK1	[65]
2	miR-30a	Renal cell carcinoma	Beclin1	Negatively regulates Beclin1 and inhibits autophagy	[66]
3	miR-181	Gastric cancer	ATG5	Impairs autophagosome formation by downregulating ATG5	[67]
4	miR-20	Breast cancer	FIP200	ULK1 complex formation is impaired by suppressing FIP200	[68]
5	miR-183	Colorectal cancer	UVRAG	Overexpression of miR-183 inhibits UVRAG, which is needed for autophagy initiation	[69]
6	miR-224-3p	Glioblastoma	ATG5, FIP200	Downregulates the expression of ATG5 and FIP200	[70]
7	miR-34c-5p	Cervical cancer	ATG4B	Negatively regulates ATG4B, which is necessary for autophagosome formation	[71]
8	miR-93	Pediatric leukemia	Beclin1	Impairs autophagy by downregulating Beclin1 expression	[72]
9	miR-124	Retinoblastoma	STX17	Directly targets and suppresses STX17, which aids in the fusion of lysosomes with autophagosomes	[73]
10	miR-1	Non-small cell lung cancer	ATG3	Abolish autophagy by downregulating ATG3 involved in conjugation machinery	[74]
11	miR-423-5p	Hepatocellular carcinoma	Not identified in the study	Promotes autophagy by increasing ATG7 and LC3-II levels	[61]
		Gastric cancer	BIM	Downregulates BIM, a negative regulator of Beclin1, and promotes autophagy	[63]
12	miR-409-5p	Ovarian cancer	FIP200	Attenuates autophagy by inhibiting FIP200	[64]

**Table 4:** The miRNAs implicated in apoptosis.

No	miRNA	Cancer	Target	Action	Reference
1	miR-16	Breast cancer	BCL-2	Abrogates the antiapoptotic effect of BCL-2 by downregulating it	[81]
2	miR-137	Ovarian cancer	XIAP	Negatively regulates XIAP, a caspase inhibitor	[82]
3	miR-345	Pancreatic cancer	BCL-2	Promotes apoptosis by suppressing the antiapoptotic protein BCL-2	[83]
4	miR-488	Osteosarcoma	BIM	Impairs apoptosis by targeting the apoptosis mediator BIM	[84]
5	miR-96	Papillary thyroid carcinoma	FOXO1/BIM axis	Indirect apoptosis suppression by negatively regulating FOXO1, as BIM is involved in downstream signalling of AKT/FOXO1 pathway	[85]
6	miR-101	Hepatocellular carcinoma	MCL-1	Promotes apoptosis by inhibiting antiapoptotic protein MCL-1	[86]
7	miR-365	Cutaneous squamous cell carcinoma	BAX	Impairs apoptosis by downregulating pro-apoptotic protein BAX	[87]
8	miR-149-5p	Acute myeloid leukemia	FASLG (Fas ligand)	Abrogates extrinsic apoptosis by negatively regulating FASLG	[88]
9	miR-199	Acute myeloid leukemia	CASP3 (caspase 3)	Impairs apoptosis by downregulating caspase 3	[89]
10	miR-675	Gastric cancer	FADD	Inhibits apoptosis by negatively regulating FADD	[90]
11	miR-224	Breast cancer	CASP9	Directly targets and impairs CASP9, attenuating apoptosis	[78]
12	miR-CHA1	Non-small cell lung cancer	XIAP	Promotes apoptosis by downregulating XIAP	[79]
13	miR-484	Non-small cell lung cancer	APAF1	Impairs apoptosis by targeting APAF1	[80]

**Table 5:** The miRNAs implicated in senescence.

No	miRNA	Cancer	Target Gene	Action	Source
1	miR-130b~301b cluster	Prostate cancer	CDKN1A, CDKN1B, CDKN2A	Promotes cellular senescence by upregulating the expression of CDK inhibitors such as CDKN1A, CDKN1B and CDKN2A	[96]
2	miR-126	B Cell Precursor Acute Lymphoblastic Leukemia (B-ALL)	P53-dependent pathway	Evades senescence by reducing the activity of p53 via targeting various p53 upstream or downstream regulators	[97]
3	miR-132	Gastric cancer	pRB	Abrogates senescence by negatively regulating pRB	[98]
4	miR-106b	Gastric cancer	CDKN1A (p21)	Impairs cellular senescence by negatively regulating CDKN1A	[99]
5	miR-494-5p	Oral squamous carcinoma	Bmi-1	Inhibits cellular senescence by suppressing Bmi-1	[100]
6	miR-203	Cervical cancer	KLF4/Survivin/p21	KLF4 induces miR-203 expression which inhibits survivin and upregulates p21, thereby inducing senescence	[101]
7	miR-137	Pancreatic cancer	KDM4A(lysine demethylase 4A)/p53/pRB	miR-137 induces pRB expression and inhibits KDM4A, a negative regulator of p53, thereby inducing senescence	[102]
8	miR-34a	Non-small cell lung cancer (NSCLC)	c-MYC	Promotes senescence by negatively regulating c-MYC, an oncogene	[103]
9	miR-128	Glioma	Bmi-1	Promotes senescence by downregulating Bmi-1	[94]
10	miR-30	Osteosarcoma	CHD7 TNRC6A	Evades senescence by downregulating CHD7 (cotranscriptional activator of p16) and TNRC6A (player in p53 activation)	[95]

**Table 6:** The miRNA implicated in vascularization.

No	miRNA	Cancer	Target	Action	Reference
1	miR-124-3p	Glioblastoma	NRP-1, transcriptional	Overexpression leads to the attenuation of angiogenesis	[119]
2	miR-526b/miR-655	Breast cancer	PTEN tumor suppressor, transcriptional	Overexpression improved angiogenesis suggesting roles as oncomiR via PTEN-regulated HIF1-α pathway	[120]
3	miR-9	Nasopharyngeal Carcinoma	MDK, exosomal secretion	Suppression of miR-9 in patient suggest its role as oncomiR. Overexpression attenuated tubal formation HUVECs	[121]
4	miR-205	Ovarian Cancer	PTEN tumor suppressor, exosomal secretion	Treatment of HUVECs with miR-205 exosome leads to an increase in tubal formation	[122]
5	miR-6868-5p	Colorectal Cancer	FOXM1, transcriptional	Overexpression leads to the reduction in endothelial tubal formation	[123]
6	miR-143-3p	Gallbladder Carcinoma	ITGA6, transcriptional	Suppression was observed in bad overall survival patients. Overexpression leads to increased tubal formation	[124]
7	miR-130b	Prostate cancer	TNF-α, transcriptional	Inhibition leads to attenuation of VEGFA, a downstream target of TNF-α suppressing angiogenesis	[125]
8	mR-23a	Nasopharyngeal Carcinoma	TSGA10, exosomal secretion	Exosomal overexpression enhanced angiogenesis	[126]
9	miR-21	Renal cell carcinoma	PCD4, proteomal	Inhibition of miR-21 attenuated MMP levels, besides inhibiting angiogenesis	[127]
10	miR-574-5p	Gastric Cancer Cells	PTPN3 proteomal	Binds to PTPN3, enhancing ERK/JNK activity and driving angiogenesis	[128]
11	miR-27a	Pancreatic Cancer	BTG2, Exosomal	miR-27a was highly expressed in cancer tissue. Exosomal mir-27a stimulates HMVEC tubal formation.	[129]
12	miR-155	Gastric Carcinoma	C-MYB/, Exosomal	Stimulates VEGF expression, leading to enhanced angiogenesis observed on HUVEC	[130]
13	miR-183-5p	Colorectal Cancer	FOXO1, Exosomal	CRC-derived- exosome enhanced tubal formation of HMEC-1 cells	[131]
14	miR-619-5p	Non-Small Cell Lung Cancer	RCAN1.4, Exosomal	Mimic transfection and leads to the increase in HUVEC tube length and tube abundance	[132]
15	miR-3064-5p	Hepatocellular carcinoma	FOXA1, transcriptional	Overexpression improves overall survival of mice and reduces tumor size; angiogenic factor suppression observed	[133]
16	miR-141	Pancreatic cancer	TM5SF1 transcriptional	Angiogenic factors were induced following inhibition of miR-141	[134]
17	miR-195	Squamous cell lung cancer	VEGF transcriptional	miRNA-195 attenuates tubal formation	[135]
18	miR-136	Gall Bladder cancer	MAP2K4 transcriptional	Mimic treatment resulted in activation of angiopoiesis	[136]
19	miR-302	Chronic Myeloid leukemia	VEGFA, secretome	Low expression was associated with bad OS. Treatment of K562 media on HUVECS attenuate capillary formation	[137]
20	miR-148a miR-30	Glioblastoma	FIH1	Regulates HIF1-α via binding directly to its inhibitor FIH1 and attenuating vascularization	[116]
21	miR-29b	Breast cancer	AKT3	Overexpression resulted in the attenuation of vascularization by downregulating AKT3, which is crucial for VEGF activation	[138]
22	miR-140-5p	Breast cancer	VEGFA	Abrogates vascularization by binding and attenuating VEGFA	[139]
23	miR-1	Gastric cancer	VEGFA	Inhibition of miR-1 leads to accumulation of VEGFA	[140]
24	miR-30d	Prostate cancer	MYPT1	Downregulation resulted in the attenuation of angiogenesis, leading to reduction in endothelial capillary tube formation	[141]
25	miR-210	Hepatocellular carcinoma	SMAD4, STAT6	Promote angiogenesis by inhibiting SMAD4 and STAT6	[118]

**Table 7:** Adhering factors found on cell junctions and their implications in metastasis.

Cell Junctions	Adhering Factors	Implication in Cancer Metastases	Reference
Gap Junction	Connexin 43	Brain cancer cells secrete cGAMP to astrocytes via connexion 43 channels, leading to STAT1-NF-κB-mediated metastasis	[152]
	Zonula Occluden (ZO-1)	ZO-1 was downregulated following overexpression of upstream regulator ZIP4 expression. This induced tumor migration	[153]
Tight Junction	Claudin	Crucial for cell anchorage. Breast cancer cells with high Claudin-2 have higher liver metastatic potential	[154]
	Occludin	Occludin upregulation suppresses metastatic potential of squamous cell carcinoma	[155]
Adherens Junction	Cadherins	Deletion of e-cadherin results in the development of both local and distant metastasis	[156]
	Catenins	Β-catenin nuclear localization is crucial for ZEB1 transcriptional activation, which negatively regulates metastasis	[157]
Desmosomes	Desmoglein 2	Loss of desmoglein enhances tumor invasiveness and migration	[158]
	Armadillo repeat units containing proteins (ARM)/plakoglobin	It was found that inhibition of proteins with the ARM structure and plakoglobin enhances the metastatic ability of bladder cancer and lung cancer, respectively	[159,160]
	Desmocollin	Desmocollin 3 downregulation leads to Akt pathway activation and decreases e-cadherin abundance in colorectal cancer, thereby enhancing metastatic potential.	[161]

**Table 8:** The miRNAs implicated in invasion and metastasis.

No	miRNA	Cancer	Target	Action	Reference
1	miR-501-3p	Hepatocellular Carcinoma	LIN7A	Metastatic cell line downregulates miR-501-3p. Overexpression of the miR inhibits metastases and EMT	[164]
2	miR-204-5p	Breast cancer	PIK3CB	miR was found to be downregulated in tumor samples. Overexpression of miR leads to metastatic attenuation in mice	[165]
3	miR-30a	Hepatocellular Carcinoma	Beclin1 and ATG5	miR-30a mediates anoikis (detachment mediated cell death) by inhibiting Beclin1 and Atg5; however, the loss of miR-30a expression in HCC leads to EMT and metastases	[166]
4	miR-193a-3p/miR-210-3p/miR5100	Bone Marrow Mesenchymal Stem cells	Exosomal miRNA targeting breast cancer metastases	Exosomal miRNA secretion of BMSC influences breast cancer metastatic potential	[167]
5	miR-466	Prostate cancer	RUNX2	Inhibition of miR-466 leads to tumorigenic properties and enhances bone metastases via RUNX2 accumulation	[168]
6	miR-203	Melanoma	SLUG	Poor OS of patients with low miR-203 expression. miR-203 overexpression leads to attenuation of early and late metastases	[169]
7	miR-103	Hepatocellular Carcinoma	VE-cadherin/ZO-1	Exosomal secretion of miR103 by HC attenuated the tight junction, resulting in an increase in metastatic potential	[170]
8	miR-103	Colorectal cancer	Zonula Occludin-1 (ZO-1)	miR-103 binds directly to the 3′-UTR of ZO-1, suggesting its role in metastases via targeting the gap junction factor	[171]
9	miR-10a	Breast cancer	Affect EMT via e- cadherin/vimentin	miR-10a suppression inhibited vimentin, disrupting the EMT pathway	[172]
10	miR-21	Breast Cancer	LZTFL1	Tumor removal via surgery reduces miR-21 expression. Suppression of miR-21 leads to attenuation of metastases; overexpression mediates metastasis in vivo	[173]
11	miR-338-3p	Ovarian cancer cells	MACC1	miR-338-3p induces metastasis via inhibiting MACC1 expression	[174]
12	miR-27b	Colorectal cancer	BTBD7	miR-27b controls the post-metastatic process via binding to BTBD7	[175]
13	miR-30 family (miR-30a, miR-30b, miR-30c, miR-30d, miR-30e)	Breast cancer	IL8, IL11, DKK-1, RUNX2, CDH11, CTGF, ITGA5, ITGB3	Overexpression of miR abrogates bone invasion and osteomimicry	[162]
14	miR-181a	Breast cancer	BAX	Downregulation of miR leads to metastasis inhibition	[176]
15	miR-1296	Hepatocellular carcinoma	SRPK1	Results in metastasis attenuation by downregulating Akt, a downstream effector of SRPK1	[177]

**Table 9:** The miRNAs implicated in metabolic rewiring.

No	miRNA	Cancer	Target	Action	Reference
1	miR-98	Colon cancer	Hexokinase 2 (HK2)	Impairs aerobic glycolysis by inhibiting glycolytic enzyme hexokinase 2	[201]
2	miR-145	Bladder cancer	KLF4	Negatively regulates KLF4, a transcriptional activator of PTBP1 that regulates PKM2, which contributes to the Warburg effect	[202]
3	miR-199a	Hepatocellular carcinoma	HIF-1a	Suppresses Warburg effect by inhibiting HIF-1a	[203]
4	miR-323a-5p	Osteosarcoma	Lactate dehydrogenase A (LDHA)	Disrupts glycolytic pathway through the inhibition of LDHA	[204]
5	miR-153	Glioblastoma	Glutaminase (GLS)	Abrogates glutamine utilization by downregulating GLS	[205]
6	miR-186	Gastric cancer	HIF-1a	Inhibits aerobic glycolysis by negatively regulating HIF-1a	[206]
7	miR-105	Breast cancer	Max-interacting protein 1 (MAXI1)/MYC	MAXI1, a transcriptional repressor of MYC, is inhibited by miR-105, thereby enhancing glucose and glutamine metabolism	[207]
8	miR-181a-5p	Non-small cell lung cancer	Acyl-CoA synthetase long-chain family member 4 (ACSL4),Sirtuin 1 (SIRT1)	Impairs lipid metabolism by inhibiting ACSL4 and abrogates glucose metabolism by suppressing SIRT1, a negative regulator of p53	[208]
9	miR-135	Pancreatic ductal adenocarcinoma (PDAC)	Phosphofructokinase-1 (PFK1)	Targets and downregulates glycolytic enzyme PFK1 and impairs aerobic glycolysis	[209]
10	miR-885-5p	Hepatocellular carcinoma	Hexokinase 2 (HK2)	Attenuates the Warburg effect by downregulating HK2	[210]

**Table 10:** The miRNAs implicated in the tumor microenvironment.

No	miRNA	Cancer	Target	Action	Reference
Cancer-associated fibroblasts (CAFs)					
1	miR-382-5p	Oral squamous cell carcinoma (OSCC)	Target not identified in study	Promotes the migration and invasion capabilities of OSCC	[214]
2	miR-34a-5p	Oral squamous cell carcinoma (OSCC)	AXL	Represses OCSS proliferation and metastasis	[215]
3	miR-139	Gastric cancer	Matrix metalloproteinase 11 (MMP11)	Represses the progression and development of metastasis of gastric cancer by modulating the level of MMP11	[216]
4	miR-214	Gastric cancer	FGF9	miR-214 is downregulated in CAFs of GC. Mimetics leads to expression of E-cadherin and suppression of Vimentin, N-cadherin and Snail, denoting repression of EMT of GC cells	[217]
5	miR-124	Ovarian cancer	Sphingosine kinase 1 (SPHK1)	Downregulates α-SMA and FAP expression to arrest cellular motility	[218]
6	miR-141-3p	Gastric cancer	STAT4	Inhibits the migration and invasion of gastric cancer and suppresses the conversion of normal fibroblasts and BMSC into CAFs by targeting regulation of the STAT4/wnt/β-catenin pathway	[219]
7	miR-21	Hepatocellular carcinoma (HCC)	PTEN	Activates the PDK1/AKT pathway in hepatic stellate cells	[224]
8	miR-3188	Head and neck cancer (HNC)	B cell lymphoma 2 (BCL2)	Regulates the proliferation and apoptosis of HNC by targeting BCL2 in vitro and in vivo.	[225]
9	miR-29b	Ovarian cancer (SKOV-3 cells)	MMP-2	Remodels the extracellular matrix and induces changes to cellular motility	[226]
10	miR-330-5p	Breast cancer	Pyruvate Kinase M1/M2 (PKM)	Represses glycolysis metabolism and cell proliferation	[227]
11	miR-125b	Breast cancer (4Ti and 4TO7)	TP53 and TP53INP1	Enhances the levels of multiple CAFs markers in resident fibroblasts leading to activation of CAF phenotypes	[228]
12	miR-27a	Gastric cancer	CSRP2	Transform fibroblasts into CAFs and enhances proliferation, motility and metastasis of tumor cells in vitro and in vivo	[229]
13	miR-1247-3p	Hepatocellular carcinoma (HCC)	B4GALT3	Activates the β1-integrin-NF-κB signaling in fibroblasts for conversion into CAFs, which secrete pro-inflammatory cytokines to enhance tumor progression	[230]
14	miR-196a	Head and neck cancer (HNC)	CDKN1B and ING5	Confers cisplatin resistance to HNC	[231]
Hypoxia					
15	miR-590-5p	Colorectal cancer (CRC)	RECK	Enhances invasive and migratory ability of cancer cells by activating matrix metalloproteinases (MMPs) and filopodia protrusions	[220]
16	miR-196-5p	Hepatocellular carcinoma (HCC)	High-mobility group AT-hook 2 (HMGA2)	Regulates the expression of HMGA2 for the proliferation and metastasis of HCC	[221]
17	miR-10b-3p	Esophageal squamous cell carcinoma (ESCC)	Testis specific 10 (TSGA10)	Increases the proliferation, migration and invasion of ESCC in both in vitro and in vivo models	[232]
18	miR-210	Pancreatic cancer (PANC-1)	HOXA9	Suppresses levels of HOXA9 to activate NFκB pathway, which drives EMT in pancreatic cancer	[222]
19	miR-210	Prostate cancer	Neural cell adhesion molecule (NCAM)	Is induced by hypoxia and functions to regulate NCAM for the progression of prostate cancer	[223]
20	miR-885-5p	Hepatocellular carcinoma (HCC)	Hexokinase 2 (HK2)	Regulates the glycometabolic activity of cancer cells via suppression of glycolytic enzymes	[210]

**Table 11:** The miRNAs implicated in immune modulation.

No	miRNA	Cancer	Target	Action	Reference
Tumor-associated macrophages (TAMs)					
1	miR-1246	Colon cancer	Upregulated CCL2, ADAM12, MMP2, CCL7Downregulated IL17A, IL7R, LEF1, S1PR1, BCL2, CD96	Reprograms macrophages to an anti-inflammatory, immunosuppressive state	[240]
2	miR-21	Lung carcinoma	IL-12	Macrophage-mediated enhancement of T cell cytotoxicity via expression of cytokines and chemokines	[239]
3	miR-29a-3p and miR-21-5p	Epithelial ovarian cancer (EOC) and TAMs	STAT3	Inhibits expression of STAT3 in CD4^+^ T cells and induces imbalance of Tregs/Th17 cell regulation	[241]
4	miR-148b	Hepatocellular carcinoma (HCC)	Colony-stimulating factor 1 (CSF1)	Progressive growth and metastasis of HCC via CSF1/CSF1 receptor-mediated TAM infiltration	[242]
5	miR-107	Gastric cancer	DICER1 and PTEN	Regulates MDSC proliferation and activation of the PI3K pathway	[243]
6	miR-130b	Spasmolytic polypeptide-expressing metaplasia (SPEM)	Cylindromatosis (Cyld) gene	Induces NFκb activity and suppresses T cell proliferation	[244]
7	miR-99b, miR-100, miR-125a, miR-125b, miR-146a, miR-146b, miR-155, let-7e	Melanomas	TLR4, SHIP-1, PTEN, SOCS1, Lin28A	Myeloid cell differentiation and polarization through pathways linked to tumor-associated immunosuppression	[245]
8	miR-10a, miR-21	Glioma	RAR-related orphan receptor alpha (RORA), phosphatase and tensin homolog (PTEN)	Initiation of MDSC-induced immunosuppressive microenvironment	[246]
Natural killer (NK) cells					
9	miR-20a	Colorectal cancer (CRC)	NKG2D ligand Major Histocompatibility Complex (MHC) class-I-related chain gene A (MICA)	Downregulates NKG2D ligand MICA levels in CRC, which promotes CRC proliferation	[247]
10	miR-130a	Non-small cell lung cancer (NSCLC)	STAT3	Increases the cytotoxic capability of NK cells by targeting STAT3 of NSCLC cells	[248]
11	miR-218-5p	Lung adenocarcinoma (LA)	Serine hydroxymethyl transferase 1 (SHMT1)	Downregulates IL-2-induced cytokine levels and cytotoxicity of NK towards LA	[249]
12	miRNA-23a, miRNA-24a, miRNA-27a	Melanoma	Ubiquitin modifiers A20, Cbl-b, and Itch	Activation of NF-κB and AP-1 via TRAF6	[250]
13	miR-561-5p	Hepatocellular carcinoma (HCC)	CX_3_CL1	Attenuate anticancer activity of CX3CG1+ NK cells via downregulation of CX3CL1	[251]
T cells					
14	miR-24-3p	Nasopharyngeal carcinoma (NPC)	FGF11	Upregulates P-ERK, P-STAT1 and P-STAT3 levels and downregulates P-STAT5 levels during T cell propagation and differentiation	[252]
15	miR-140	Gastric cancers	PD-L1	Overexpression leads to increase in cytotoxic CD8+ T cells and reductions in MDSC and Tregs in the immediate tumor microenvironment	[253]
16	miR-149-3p	Breast tumor	T cell inhibitor receptors PD-1, TIM-3, BTLA and Foxp1	Increase T cell inhibitor receptor expression, reduction of apoptosis and secretion of effector cytokines, including IL-2, TNF-α and IFN-γ	[254]
17	miR-448	Colon cancer	Indoleamine 2,3-dioxygenase 1 (IDO1)	Regulates posttranscriptional levels of IDO1 protein and mRNA, inhibits apoptosis of CD8^+^ T cells by reducing IDO1 enzyme activity	[255]
18	miR-195	Lung adenocarcinoma	CCDC88C/Wnt	Regulates CCDC88C expression for CD4^+^ T cell activation	[256]

**Table 12:** The miRNA exhibiting an overlap function.

No	miRNA	Overlap Hallmark	Cancer Types	References
1	miR-132	i. Selective Proliferative Advantage ii. Altered Stress Response-Senescence	Ovarian cancer Gastric cancer	[22] [98]
2	miR-183	i. Selective Proliferative Advantage ii. Altered Stress Response-Autophagy iii. Vascularization	Breast cancer Colorectal cancer Colorectal cancer	[26] [69] [131]
3	miR-141	i. Selective Proliferative Advantage ii. Vascularization iii. Tumor microenvironment	Nasopharyngeal carcinoma Pancreatic cancer Gastric cancer	[28] [134] [219]
4	miR-136	i. Selective Proliferative Advantage ii. Vascularization	Prostate cancer Gall bladder cancer	[29] [136]
5	miR-124	i. Selective Proliferative Advantage ii. Altered Stress Response- Autophagy iii. Vascularization iv. Tumor microenvironment	Breast cancer Retinoblastoma Glioblastoma Ovarian cancer	[30] [73] [119] [218]
6	miR-93	i. Selective Proliferative Advantage ii. Altered Stress Response-Autophagy	Osteosarcoma Leukemia	[60] [72]
7	miR-196a	i. Selective Proliferative Advantage ii. Tumor microenvironment	Laryngeal cancer Head and neck cancer	[34] [231]
8	miR-145	i. Altered stress response-DNA repair ii. Metabolic rewiring	Colorectal cancer Bladder cancer	[52] [202]
9	miR-205	i. Altered stress response-DNA repair ii. Vascularization	Head and neck squamous cell carcinoma Ovarian cancer	[54] [122]
10	miR-338	i. Altered stress response- DNA repair ii. Invasion and Metastasis	Glioblastoma Ovarian cancer	[58] [174]
11	miR-30a	i. Altered stress response-Autophagy ii. Invasion and Metastasis	Renal cell carcinoma Hepatocellular carcinoma	[66] [166]
12	miR-181	i. Altered stress response-Autophagy ii. Metabolic rewiring iii. Invasion and Metastasis	Gastric cancer Non-small cell lung cancer Breast cancer	[67] [208] [176]
13	miR-20	i. Selective proliferative advantage ii. Altered stress response-Autophagy iii. Immune modulation	Multiple myeloma Breast cancer Colorectal cancer	[19] [96] [247]
14	miR-224	i. Altered stress response-Autophagy ii. Altered stress response-Apoptosis	Glioblastoma Breast cancer	[70] [78]
15	miR-1	i. Altered stress response-Autophagy ii. Vascularization	Non-small cell lung cancer Gastric cancer	[74] [140]
16	miR-137	i. Altered stress response-Apoptosis ii. Altered stress response-Senescence	Ovarian cancer Pancreatic cancer	[82] [102]
17	miR-101	i. Selective proliferative advantage ii. Altered stress response-Apoptosis	Diffuse large B cell lymphoma Hepatocellular carcinoma	[18] [86]
18	miR-149	i. Altered stress response-Apoptosis ii. Immune modulation	Acute myeloid leukemia Breast cancer	[88] [254]
19	miR-199	i. Altered stress response-Apoptosis ii. Metabolic rewiring	Acute myeloid leukemia Hepatocellular carcinoma	[89] [203]
20	miR-130b	i. Altered stress response-Senescence ii. Vascularization iii. Immune modulation	Prostate cancer Prostate cancer Spasmolytic polypeptide-expressing metaplasia (SPEM)	[96] [125] [244]
21	miR-203	i. Altered stress response- DNA repair ii. Altered stress response-Senescence iii. Invasion and Metastasis	Ovarian cancer Cervical cancer Melanoma	[48] [101] [169]
22	miR-34a	i. Altered stress response-Senescence ii. Tumor microenvironment	Non-small cell lung cancer Oral squamous cell carcinoma	[103] [215]
23	miR-21	i. Vascularization ii. Invasion and Metastasis iii. Tumor microenvironment iv. Immune modulation	Renal cell carcinoma Breast cancer Hepatocellular carcinoma Lung carcinoma	[127] [173] [224] [239]
24	miR-27a	i. Vascularization ii. Tumor microenvironment iii. Immune modulation	Pancreatic cancer Gastric cancer Melanoma	[129] [229] [250]
25	miR-155	i. Vascularization ii. Immune modulation	Gastric carcinoma Melanoma	[155] [245]
26	miR-195	i. Vascularization ii. Invasion and Metastasis iii. Immune modulation	Squamous cell lung cancer Breast cancer Lung adenocarcinoma	[135] [163] [256]
27	miR-210	i. Vascularization ii. Invasion and Metastasis iii. Tumor microenvironment	Hepatocellular carcinoma Bone Marrow Mesenchymal Stem cells Pancreatic cancer Prostate cancer	[118] [167] [222] [223]
28	miR-10a	i. Invasion and Metastasis ii. Immune modulation	Breast cancer Glioma	[172] [246]
29	miR-29b	i. Vascularization ii. Tumor microenvironment	Breast cancer Ovarian cancer	[138] [226]
30	miR-125b	i. Tumor microenvironment ii. Immune modulation	Breast cancer Melanoma	[228] [245]
31	miR-590	i. Selective Proliferative Advantage ii. Tumor microenvironment	T cell acute lymphoblastic leukemia Colorectal cancer	[20] [220]
32	miR-140	i. Vascularization ii. Immune modulation	Breast cancer Gastric cancer	[107] [253]
33	miR-885-5p	i. Metabolic rewiring ii. Tumor microenvironment	Hepatocellular carcinoma	[210]

## Data Availability

Not applicable.

## References

[1] Hanahan D., Weinberg R.A. (2000). The Hallmarks of Cancer. Cell.

[2] Hanahan D., Weinberg R.A. (2011). Hallmarks of cancer: The next generation. Cell.

[3] Fouad Y.A., Aanei C. (2017). Revisiting the hallmarks of cancer. Am. J. Cancer Res..

[4] Catalanotto C., Cogoni C., Zardo G. (2016). MicroRNA in Control of Gene Expression: An Overview of Nuclear Functions. Int. J. Mol. Sci..

[5] Van Roosbroeck K., Calin G.A. (2017). Cancer Hallmarks and MicroRNAs: The Therapeutic Connection. Adv. Cancer Res..

[6] Stavast C., Erkeland S. (2019). The Non-Canonical Aspects of MicroRNAs: Many Roads to Gene Regulation. Cells.

[7] Manasa V., Kannan S. (2017). Impact of microRNA dynamics on cancer hallmarks: An oral cancer scenario. Tumor Biol..

[8] Tan W., Liu B., Qu S., Liang G., Luo W., Gong C. (2017). MicroRNAs and cancer: Key paradigms in molecular therapy (Review). Oncol. Lett..

[9] Peng Y., Croce C.M. (2016). The role of MicroRNAs in human cancer. Signal Transduct. Target. Ther..

[10] Feitelson M.A., Arzumanyan A., Kulathinal R.J., Blain S.W., Holcombe R.F., Mahajna J., Marino M., Chantar M.L.M., Nawroth R., Sanchez-Garcia I. (2015). Sustained proliferation in cancer: Mechanisms and novel therapeutic targets. Semin. Cancer Biol..

[11] Wee P., Wang Z. (2017). Epidermal Growth Factor Receptor Cell Proliferation Signaling Pathways. Cancers.

[12] Nogués L., Palacios-García J., Reglero C., Rivas V., Neves M., Ribas C., Penela P., Mayor F. (2018). G protein-coupled receptor kinases (GRKs) in tumorigenesis and cancer progression: GPCR regulators and signaling hubs. Semin. Cancer Biol..

[13] Dienstmann R., Rodon J., Serra V., Tabernero J. (2014). Picking the Point of Inhibition: A Comparative Review of PI3K/AKT/mTOR Pathway Inhibitors. Mol. Cancer Ther..

[14] Singh S.S., Yap W.N., Arfuso F., Kar S., Wang C., Cai W., Dharmarajan A.M., Sethi G., Kumar A.P. (2015). Targeting the PI3K/Akt signaling pathway in gastric carcinoma: A reality for personalized medicine?. World J. Gastroenterol..

[15] Peng Q., Deng Z., Pan H., Gu L., Liu O., Tang Z. (2018). Mitogen-activated protein kinase signaling pathway in oral cancer. Oncol. Lett..

[16] Tomiyama A., Ichimura K. (2019). Signal transduction pathways and resistance to targeted therapies in glioma. Semin. Cancer Biol..

[17] Otto T., Sicinski P. (2017). Cell cycle proteins as promising targets in cancer therapy. Nat. Rev. Cancer.

[18] Huang Y., Zou Y., Lin L., Ma X., Zheng R. (2019). miR-101 regulates the cell proliferation and apoptosis in diffuse large B-cell lymphoma by targeting MEK1 via regulation of the ERK/MAPK signaling pathway. Oncol. Rep..

[19] Jiang Y., Chang H., Chen G. (2018). Effects of microRNA 20a on the proliferation, migration and apoptosis of multiple myeloma via the PTEN/PI3K/AKT signaling pathway. Oncol. Lett..

[20] Miao M.-H., Ji X.-Q., Zhang H., Xu J., Zhu H., Shao X.-J. (2016). miR-590 promotes cell proliferation and invasion in T-cell acute lymphoblastic leukaemia by inhibiting RB1. Oncotarget.

[21] Li J., Hu L., Tian C., Lu F., Wu J., Liu L. (2015). microRNA-150 promotes cervical cancer cell growth and survival by targeting FOXO4. BMC Mol. Biol..

[22] Tian H., Hou L., Xiong Y.-M., Huang J.-X., Zhang W.-H., Pan Y.-Y., Song X.-R. (2016). miR-132 targeting E2F5 suppresses cell proliferation, invasion, migration in ovarian cancer cells. Am. J. Transl. Res..

[23] Li Q., Qiu X.-M., Li Q.-H., Wang X.-Y., Li L., Xu M., Dong M., Xiao Y.-B. (2015). MicroRNA-424 may function as a tumor suppressor in endometrial carcinoma cells by targeting E2F7. Oncol. Rep..

[24] Chen G., Fang T., Huang Z., Qi Y., Du S., Di T., Lei Z., Zhang X., Yan W. (2016). MicroRNA-133a Inhibits Osteosarcoma Cells Proliferation and Invasion via Targeting IGF-1R. Cell. Physiol. Biochem..

[25] Zhang W., Liu K., Liu S., Ji B., Wang Y., Liu Y. (2015). MicroRNA-133a functions as a tumor suppressor by targeting IGF-1R in hepatocellular carcinoma. Tumor Biol..

[26] Cheng Y., Xiang G., Meng Y., Dong R. (2016). MiRNA-183-5p promotes cell proliferation and inhibits apoptosis in human breast cancer by targeting the PDCD4. Reprod. Biol..

[27] Xiao Y., Deng T., Su C., Shang Z. (2017). MicroRNA 217 inhibits cell proliferation and enhances chemosensitivity to doxorubicin in acute myeloid leukemia by targeting KRAS. Oncol. Lett..

[28] Liu Y., Zhao R., Wang H., Luo Y., Wang X., Niu W., Zhou Y., Wen Q., Fan S., Li X. (2016). miR-141 is involved in BRD7-mediated cell proliferation and tumor formation through suppression of the PTEN/AKT pathway in nasopharyngeal carcinoma. Cell Death Dis..

[29] Zhu Y., Shao S., Pan H., Cheng Z., Rui X. (2018). MicroRNA-136 inhibits prostate cancer cell proliferation and invasion by directly targeting mitogen-activated protein kinase kinase 4. Mol. Med. Rep..

[30] Shi P., Chen C., Li X., Wei Z., Liu Z., Liu Y. (2019). MicroRNA-124 suppresses cell proliferation and invasion of triple negative breast cancer cells by targeting STAT3. Mol. Med. Rep..

[31] Jiang L., Yang W., Bian W., Yang H., Wu X., Li Y., Feng W., Liu X. (2018). MicroRNA-623 Targets Cyclin D1 to Inhibit Cell Proliferation and Enhance the Chemosensitivity of Cells to 5-Fluorouracil in Gastric Cancer. Oncol. Res. Featur. Preclin. Clin..

[32] Moradimotlagh A., Arefian E., Valojerdi R.R., Ghaemi S., Adegani F.J., Soleimani M. (2020). MicroRNA-129 Inhibits Glioma Cell Growth by Targeting CDK4, CDK6, and MDM2. Mol. Ther.-Nucleic Acids.

[33] He Y., Yu B. (2017). MicroRNA-93 promotes cell proliferation by directly targeting P21 in osteosarcoma cells. Exp. Ther. Med..

[34] Jin C., Zhang Y., Li J. (2016). Upregulation of MiR-196a promotes cell proliferation by downregulating p27kip1 in laryngeal cancer. Biol. Res..

[35] Ye C.-Y., Zheng C.-P., Ying W.-W., Weng S.-S. (2018). Up-regulation of microRNA-497 inhibits the proliferation, migration and invasion but increases the apoptosis of multiple myeloma cells through the MAPK/ERK signaling pathway by targeting Raf-1. Cell Cycle.

[36] Zhang C., Wang H., Liu X., Hu Y., Ding L., Zhang X., Sun Q., Li Y. (2019). Oncogenic microRNA-411 promotes lung carcinogenesis by directly targeting suppressor genes SPRY4 and TXNIP. Oncogene.

[37] Pfeffer C.M., Singh A.T.K. (2018). Apoptosis: A Target for Anticancer Therapy. Int. J. Mol. Sci..

[38] Bian L., Meng Y., Zhang M., Li D. (2019). MRE11-RAD50-NBS1 complex alterations and DNA damage response: Implications for cancer treatment. Mol. Cancer.

[39] Syed A., Tainer J.A. (2018). The MRE11–RAD50–NBS1 Complex Conducts the Orchestration of Damage Signaling and Outcomes to Stress in DNA Replication and Repair. Annu. Rev. Biochem..

[40] Pilié P.G., Tang C., Mills G.B., Yap T.A. (2019). State-of-the-art strategies for targeting the DNA damage response in cancer. Nat. Rev. Clin. Oncol..

[41] Tšuiko O., Jatsenko T., Grace LK P., Kurg A., Vermeesch J.R., Lanner F., Salumets A. (2019). A speculative outlook on embryonic aneuploidy: Can molecular pathways be involved?. Dev. Biol..

[42] Pascal J.M. (2018). The comings and goings of PARP-1 in response to DNA damage. DNA Repair.

[43] Ronco C., Martin A.R., Demange L., Benhida R. (2017). ATM, ATR, CHK1, CHK2 and WEE1 inhibitors in cancer and cancer stem cells. MedChemComm.

[44] He M., Zhou W., Li C., Guo M. (2016). MicroRNAs, DNA damage response, and cancer treatment. Int. J. Mol. Sci..

[45] Majidinia M., Yousefi B. (2016). DNA damage response regulation by microRNAs as a therapeutic target in cancer. DNA Repair.

[46] Lai J., Yang H., Zhu Y., Ruan M., Huang Y., Zhang Q. (2019). MiR-7-5p-mediated downregulation of PARP1 impacts DNA homologous recombination repair and resistance to doxorubicin in small cell lung cancer. BMC Cancer.

[47] Yang F., Guo L., Cao Y., Li S., Li J., Liu M. (2018). MicroRNA-7-5p Promotes Cisplatin Resistance of Cervical Cancer Cells and Modulation of Cellular Energy Homeostasis by Regulating the Expression of the PARP-1 and BCL2 Genes. Med. Sci. Monit..

[48] Liu H.-Y., Zhang Y.-Y., Zhu B.-L., Feng F.-Z., Zhang H.-T., Yan H., Zhou B. (2019). MiR-203a-3p regulates the biological behaviors of ovarian cancer cells through mediating the Akt/GSK-3β/Snail signaling pathway by targeting ATM. J. Ovarian Res..

[49] Yang H., Luo J., Liu Z., Zhou R., Luo H. (2015). MicroRNA-138 Regulates DNA Damage Response in Small Cell Lung Cancer Cells by Directly Targeting H2AX. Cancer Investig..

[50] Liao X.-H., Zheng L., He H.-P., Zheng D.-L., Wei Z.-Q., Wang N., Dong J., Ma W.-J., Zhang T.-C. (2015). STAT3 regulated ATR via microRNA-383 to control DNA damage to affect apoptosis in A431 cells. Cell. Signal..

[51] Lai T.-H., Ewald B., Zecevic A., Liu C., Sulda M., Papaioannou D., Garzon R., Blachly J.S., Plunkett W., Sampath D. (2016). HDAC Inhibition Induces MicroRNA-182, which Targets RAD51 and Impairs HR Repair to Sensitize Cells to Sapacitabine in Acute Myelogenous Leukemia. Clin. Cancer Res..

[52] Liu R.-L., Dong Y., Deng Y.-Z., Wang W.-J., Li W.-D. (2015). Tumor suppressor miR-145 reverses drug resistance by directly targeting DNA damage-related gene RAD18 in colorectal cancer. Tumor Biol..

[53] He X., Fan S. (2018). hsa-miR-212 modulates the radiosensitivity of glioma cells by targeting BRCA1. Oncol. Rep..

[54] Valenti F., Sacconi A., Ganci F., Grasso G., Strano S., Blandino G., Di Agostino S. (2019). The miR-205-5p/BRCA1/RAD17 Axis Promotes Genomic Instability in Head and Neck Squamous Cell Carcinomas. Cancers.

[55] Huang Y.-Z., Zhang J., Shao H.-Y., Chen J.-P., Zhao H.-Y. (2015). MicroRNA-191 promotes osteosarcoma cells proliferation by targeting checkpoint kinase 2. Tumor Biol..

[56] Peng D., Dong J., Zhao Y., Peng X., Tang J., Chen X., Wang L., Hu D.-N., Reinach P.S., Qu J. (2019). miR-142-3p suppresses uveal melanoma by targeting CDC25C, TGFβR1, GNAQ, WASL, and RAC1. Cancer Manag. Res..

[57] Xu S., Huang H., Chen Y.-N., Deng Y.-T., Zhang B., Xiong X.-D., Yuan Y., Zhu Y., Huang H., Xie L. (2016). DNA damage responsive miR-33b-3p promoted lung cancer cells survival and cisplatin resistance by targeting p21WAF1/CIP1. Cell Cycle.

[58] Besse A., Sana J., Lakomy R., Kren L., Fadrus P., Smrcka M., Hermanova M., Jancalek R., Reguli S., Lipina R. (2015). MiR-338-5p sensitizes glioblastoma cells to radiation through regulation of genes involved in DNA damage response. Tumor Biol..

[59] Towers C.G., Wodetzki D., Thorburn A. (2020). Autophagy and cancer: Modulation of cell death pathways and cancer cell adaptations Autophagy and cancer. J. Cell Biol..

[60] Levy J.M.M., Towers C.G., Thorburn A. (2017). Targeting autophagy in cancer. Nat. Rev. Cancer.

[61] Stiuso P., Potenza N., Lombardi A., Ferrandino I., Monaco A., Zappavigna S., Vanacore D., Mosca N., Castiello F., Porto S. (2015). MicroRNA-423-5p Promotes Autophagy in Cancer Cells and Is Increased in Serum from Hepatocarcinoma Patients Treated with Sorafenib. Mol. Ther.-Nucleic Acids.

[62] Gross A., Katz S.G. (2017). Non-apoptotic functions of BCL-2 family proteins. Cell Death Differ..

[63] Kong P., Zhu X., Geng Q., Xia L., Sun X., Chen Y., Li W., Zhou Z., Zhan Y., Xu D. (2017). The microRNA-423-3p-Bim Axis Promotes Cancer Progression and Activates Oncogenic Autophagy in Gastric Cancer. Mol. Ther..

[64] Cheng Y., Ban R., Liu W., Wang H., Li S., Yue Z., Zhu G., Zhuan Y., Wang C. (2020). MiRNA-409-3p enhances cisplatin-sensitivity of ovarian cancer cells by blocking the autophagy mediated by Fip200. Oncol. Res. Featur. Preclin. Clin. Cancer Ther..

[65] Jin F., Wang Y., Li M., Zhu Y., Liang H., Wang C., Wang F., Zhang C.-Y., Zen K., Li L. (2018). MiR-26 enhances chemosensitivity and promotes apoptosis of hepatocellular carcinoma cells through inhibiting autophagy. Cell Death Dis..

[66] Zheng B., Zhu H., Gu D., Pan X., Qian L., Xue B., Yang D., Zhou J., Shan Y. (2015). MiRNA-30a-mediated autophagy inhibition sensitizes renal cell carcinoma cells to sorafenib. Biochem. Biophys. Res. Commun..

[67] Zhao J., Nie Y., Wang H., Lin Y. (2016). miR-181a suppresses autophagy and sensitizes gastric cancer cells to cisplatin. Gene.

[68] Li S., Qiang Q., Shan H., Shi M., Gan G., Ma F., Chen B. (2016). MiR-20a and miR-20b negatively regulate autophagy by targeting RB1CC1/FIP200 in breast cancer cells. Life Sci..

[69] Huangfu L., Liang H., Wang G., Su X., Li L., Du Z., Hu M., Dong Y., Bai X., Liu T. (2015). miR-183 regulates autophagy and apoptosis in colorectal cancer through targeting of UVRAG. Oncotarget.

[70] Guo X., Xue H., Guo X., Gao X., Xu S., Yan S., Han X., Li T., Shen J., Li G. (2015). MiR224-3p inhibits hypoxia-induced autophagy by targeting autophagy-related genes in human glioblastoma cells. Oncotarget.

[71] Wu Y., Ni Z., Yan X., Dai X., Hu C., Zheng Y., He F., Lian J. (2016). Targeting the MIR34C-5p-ATG4B-autophagy axis enhances the sensitivity of cervical cancer cells to pirarubicin. Autophagy.

[72] Wu X., Feng X., Zhao X., Ma F., Liu N., Guo H., Li C., Du H., Zhang B. (2016). Role of Beclin-1-Mediated Autophagy in the Survival of Pediatric Leukemia Cells. Cell. Physiol. Biochem..

[73] Huang J., Yang Y., Fang F., Liu K. (2018). MALAT1 modulates the autophagy of retinoblastoma cell through miR-124-mediated stx17 regulation. J. Cell. Biochem..

[74] Hua L., Zhu G., Wei J. (2018). MicroRNA-1 overexpression increases chemosensitivity of non-small cell lung cancer cells by inhibiting autophagy related 3-mediated autophagy. Cell Biol. Int..

[75] Cao K., Tait S.W. (2018). Apoptosis and cancer: Force awakens, phantom menace, or both?. Int. Rev. Cell Mol. Biol..

[76] Ichim G., Tait S. (2016). A fate worse than death: Apoptosis as an oncogenic process. Nat. Rev. Cancer.

[77] Jan R., Chaudhry G.-E. (2019). Understanding Apoptosis and Apoptotic Pathways Targeted Cancer Therapeutics. Adv. Pharm. Bull..

[78] Zhang L., Zhang X., Wang X., He M., Qiao S. (2019). MicroRNA-224 Promotes Tumorigenesis through Downregulation of Caspase-9 in Triple-Negative Breast Cancer. Dis. Markers.

[79] Yoo J.K., Lee J.M., Kang S.H., Jeon S.H., Kim C.M., Oh S.-H., Kim J.K. (2019). The novel microRNA hsa-miR-CHA1 regulates cell proliferation and apoptosis in human lung cancer by targeting XIAP. Lung Cancer.

[80] Li T., Ding Z.-L., Zheng Y.-L., Wang W. (2017). MiR-484 promotes non-small-cell lung cancer (NSCLC) progression through inhibiting Apaf-1 associated with the suppression of apoptosis. Biomed. Pharmacother..

[81] Mobarra N., Shafiee A., Rad S.M.A.H., Tasharrofi N., Soufi-Zomorod M., Hafizi M., Movahed M., Kouhkan F., Soleimani M. (2015). Overexpression of microRNA-16 declines cellular growth, proliferation and induces apoptosis in human breast cancer cells. Vitr. Cell. Dev. Biol.-Anim..

[82] Li X., Chen W., Zeng W., Wan C., Duan S., Jiang S. (2017). microRNA-137 promotes apoptosis in ovarian cancer cells via the regulation of XIAP. Br. J. Cancer.

[83] Srivastava S.K., Bhardwaj A., Arora S., Tyagi N., Singh S., Andrews J., McClellan S., Wang B., Singh A. (2015). MicroRNA-345 induces apoptosis in pancreatic cancer cells through potentiation of caspase-dependent and -independent pathways. Br. J. Cancer.

[84] Zhou C., Tan W., Lv H., Gao F., Sun J. (2016). Hypoxia-inducible microRNA-488 regulates apoptosis by targeting Bim in osteosarcoma. Cell. Oncol..

[85] Song H.-M., Luo Y., Li D.-F., Wei C.-K., Hua K.-Y., Song J.-L., Xu H., Maskey N., Fang L. (2015). MicroRNA-96 plays an oncogenic role by targeting FOXO1 and regulating AKT/FOXO1/Bim pathway in papillary thyroid carcinoma cells. Int. J. Clin. Exp. Pathol..

[86] He H., Tian W., Chen H., Deng Y. (2016). MicroRNA-101 sensitizes hepatocellular carcinoma cells to doxorubicin-induced apoptosis via targeting Mcl-1. Mol. Med. Rep..

[87] Zhou L., Gao R., Wang Y., Zhou M., Ding Z. (2017). Loss of BAX by miR-365 Promotes Cutaneous Squamous Cell Carcinoma Progression by Suppressing Apoptosis. Int. J. Mol. Sci..

[88] Tian P., Yan L. (2016). Inhibition of MicroRNA-149-5p Induces Apoptosis of Acute Myeloid Leukemia Cell Line THP-1 by Targeting Fas Ligand (FASLG). Med. Sci. Monit..

[89] Xue F., Zhu Y., Xu F., Zhou L.-J., Han F., Wang S.-C. (2019). MicroRNA-199 inhibits proliferation and promotes apoptosis in children with acute myeloid leukemia by mediating caspase-3. Eur. Rev. Med. Pharmacol. Sci..

[90] Yan J., Zhang Y., She Q., Li X., Peng L., Wang X., Liu S., Shen X., Zhang W., Dong Y. (2017). Long Noncoding RNA H19/miR-675 Axis Promotes Gastric Cancer via FADD/Caspase 8/Caspase 3 Signaling Pathway. Cell. Physiol. Biochem..

[91] Mavrogonatou E., Pratsinis H., Kletsas D. (2019). The role of senescence in cancer development. Semin. Cancer Biol..

[92] Munk R., Panda A.C., Grammatikakis I., Gorospe M., Abdelmohsen K. (2017). Senescence-associated microRNAs. Int. Rev. Cell Mol. Biol..

[93] Lee S., Lee J.-S. (2019). Cellular senescence: A promising strategy for cancer therapy. BMB Rep..

[94] Sun J., Ye L., Wang C., Li N., Wang D., Li X. (2018). MicroRNA-128 increases glioma cell radio-sensitivity by suppressing senescent evasion through oncogene Bmi-1. Int. J. Clin. Exp. Pathol..

[95] Su W., Hong L., Xu X., Huang S., Herpai D., Li L., Xu Y., Truong L., Hu W.-Y., Wu X. (2018). miR-30 disrupts senescence and promotes cancer by targeting both p16INK4A and DNA damage pathways. Oncogene.

[96] Ramalho-Carvalho J., Graça I., Gomez A., Oliveira J., Henrique R., Esteller M., Jerónimo C. (2017). Downregulation of miR-130b~301b cluster is mediated by aberrant promoter methylation and impairs cellular senescence in prostate cancer. J. Hematol. Oncol..

[97] Nucera S., Giustacchini A., Boccalatte F., Calabria A., Fanciullo C., Plati T., Ranghetti A., Garcia-Manteiga J., Cittaro D., Benedicenti F. (2016). miRNA-126 Orchestrates an Oncogenic Program in B Cell Precursor Acute Lymphoblastic Leukemia. Cancer Cell.

[98] Gao F.-Y., Liu Q.-Y., Yuan L., Xuan S.-Y. (2015). Upregulation of microRNA-132 in gastric cancer promotes cell proliferation via retinoblastoma 1 targeting. Mol. Med. Rep..

[99] Dong X., Hu X., Chen J., Hu D., Chen L.-F. (2018). BRD4 regulates cellular senescence in gastric cancer cells via E2F/miR-106b/p21 axis. Cell Death Dis..

[100] Weng J.-H., Yu C.-C., Lee Y.-C., Lin C.-W., Chang W.-W., Kuo Y.-L. (2016). miR-494-3p Induces Cellular Senescence and Enhances Radiosensitivity in Human Oral Squamous Carcinoma Cells. Int. J. Mol. Sci..

[101] Xu Q., Liu M., Zhang J., Xue L., Zhang G., Hu C., Wang Z., He S., Chen L., Ma K. (2016). Overexpression of KLF4 promotes cell senescence through microRNA-203-survivin-p21 pathway. Oncotarget.

[102] Neault M., Mallette F.A., Richard S. (2016). miR-137 Modulates a Tumor Suppressor Network-Inducing Senescence in Pancreatic Cancer Cells. Cell Rep..

[103] He X., Yang A., McDonald D.G., Riemer E.C., Vanek K.N., Schulte B.A., Wang G.Y. (2017). MiR-34a modulates ionizing radiation-induced senescence in lung cancer cells. Oncotarget.

[104] Loizzi V., Del Vecchio V., Gargano G., De Liso M., Kardashi A., Naglieri E., Resta L., Cicinelli E., Cormio G. (2017). Biological Pathways Involved in Tumor Angiogenesis and Bevacizumab Based Anti-Angiogenic Therapy with Special References to Ovarian Cancer. Int. J. Mol. Sci..

[105] Dome B., Hendrix M.J., Paku S., Tóvári J., Tímár J. (2007). Alternative Vascularization Mechanisms in Cancer: Pathology and Therapeutic Implications. Am. J. Pathol..

[106] Doktorova H., Hrabeta J., Khalil M.A., Eckschlager T. (2015). Hypoxia-induced chemoresistance in cancer cells: The role of not only HIF-1. Biomed. Pap..

[107] Lu Y., Qin T., Li J., Wang L., Zhang Q., Jiang Z., Mao J. (2017). MicroRNA-140-5p inhibits invasion and angiogenesis through targeting VEGF-A in breast cancer. Cancer Gene Ther..

[108] Zimna A., Kurpisz M. (2015). Hypoxia-Inducible Factor-1 in Physiological and Pathophysiological Angiogenesis: Applications and Therapies. BioMed Res. Int..

[109] Batty M., Pugh R., Rathinam I., Simmonds J., Walker E., Forbes A., Anoopkumar-Dukie S., McDermott C.M., Spencer B., Christie D. (2016). The Role of α1-Adrenoceptor Antagonists in the Treatment of Prostate and Other Cancers. Int. J. Mol. Sci..

[110] Chang Y.-C., Chan Y.-C., Chang W.-M., Lin Y.-F., Yang C.-J., Su C.-Y., Huang M.-S., Wu A.T., Hsiao M. (2017). Feedback regulation of ALDOA activates the HIF-1α/MMP9 axis to promote lung cancer progression. Cancer Lett..

[111] Li Y.-Y., Zheng Y.-L. (2017). Hypoxia promotes invasion of retinoblastoma cells in vitro by upregulating HIF-1α/MMP9 signaling pathway. Eur. Rev. Med. Pharmacol. Sci..

[112] Oren R., Addadi S., Haziza L.N., Dafni H., Rotkopf R., Meir G., Fishman A., Neeman M. (2016). Fibroblast recruitment as a tool for ovarian cancer detection and targeted therapy. Int. J. Cancer.

[113] Webb A.H., Gao B.T., Goldsmith Z.K., Irvine A.S., Saleh N., Lee R.P., Lendermon J.B., Bheemreddy R., Zhang Q., Brennan R.C. (2017). Inhibition of MMP-2 and MMP-9 decreases cellular migration, and angiogenesis in In Vitro models of retinoblastoma. BMC Cancer.

[114] Gómez-Escudero J., Clemente C., García-Weber D., Acín-Pérez R., Millán J., Enríquez J.A., Bentley K., Carmeliet P., Arroyo A.G. (2019). PKM2 regulates endothelial cell junction dynamics and angiogenesis via ATP production. Sci. Rep..

[115] Roux Q., Gavard J. (2019). Endothelial Cell-Cell Junctions in Tumor Angiogenesis. Tumor Angiogenesis Key Target for Cancer Therapy.

[116] Wong H.-K.A., El Fatimy R., Onodera C., Wei Z., Yi M., Mohan A., Gowrisankaran S., Karmali P., Marcusson E., Wakimoto H. (2015). The Cancer Genome Atlas Analysis Predicts MicroRNA for Targeting Cancer Growth and Vascularization in Glioblastoma. Mol. Ther..

[117] Zeng Z., Li Y., Pan Y., Lan X., Song F., Sun J., Zhou K., Liu X., Ren X., Wang F. (2018). Cancer-derived exosomal miR-25-3p promotes pre-metastatic niche formation by inducing vascular permeability and angiogenesis. Nat. Commun..

[118] Lin X.-J., Fang J.-H., Yang X.-J., Zhang C., Yuan Y., Zheng L., Zhuang S.-M. (2018). Hepatocellular Carcinoma Cell-Secreted Exosomal MicroRNA-210 Promotes Angiogenesis In Vitro and In Vivo. Mol. Ther.-Nucleic Acids.

[119] Zhang G., Chen L., Khan A.A., Li B., Gu B., Lin F., Su X., Yan J. (2018). miRNA-124-3p/neuropilin-1(NRP-1) axis plays an important role in mediating glioblastoma growth and angiogenesis. Int. J. Cancer.

[120] Hunter S., Nault B., Ugwuagbo K., Maiti S., Majumder M. (2019). Mir526b and Mir655 Promote Tumour Associated Angiogenesis and Lymphangiogenesis in Breast Cancer. Cancers.

[121] Lu J., Liu Q.-H., Wang F., Tan J.-J., Deng Y.-Q., Peng X.-H., Liu X., Zhang B., Xu X., Li X.-P. (2018). Exosomal miR-9 inhibits angiogenesis by targeting MDK and regulating PDK/AKT pathway in nasopharyngeal carcinoma. J. Exp. Clin. Cancer Res..

[122] He L., Zhu W., Chen Q., Yuan Y., Wang Y., Wang J., Wu X. (2019). Ovarian cancer cell-secreted exosomal miR-205 promotes metastasis by inducing angiogenesis. Theranostics.

[123] Wang Y., Wu M., Lei Z., Huang M., Li Z., Wang L., Cao Q., Han D., Chang Y., Chen Y. (2018). Dysregulation of miR-6868-5p/FOXM1 circuit contributes to colorectal cancer angiogenesis. J. Exp. Clin. Cancer Res..

[124] Jin Y.-P., Hu Y.-P., Wu X.-S., Wu Y.-S., Ye Y.-Y., Li H.-F., Liu Y.-C., Jiang L., Liu F.-T., Zhang Y.-J. (2018). miR-143-3p targeting of ITGA6 suppresses tumour growth and angiogenesis by downregulating PLGF expression via the PI3K/AKT pathway in gallbladder carcinoma. Cell Death Dis..

[125] Mu H.Q., He Y.H., Wang S.B., Yang S., Wang Y.J., Nan C.J., Bao Y.F., Xie Q.P., Chen Y.H. (2020). MiR-130b/TNF-α/NF-κB/VEGFA loop inhibits prostate cancer angiogenesis. Clin. Transl. Oncol..

[126] Bao L., You B., Shi S., Shan Y., Zhang Q., Yue H., Zhang J., Zhang W., Shi Y., Liu Y. (2018). Metastasis-associated miR-23a from nasopharyngeal carcinoma-derived exosomes mediates angiogenesis by repressing a novel target gene TSGA10. Oncogene.

[127] Fan B., Jin Y., Zhang H., Zhao R., Sun M., Sun M., Yuan X., Wang W., Wang X., Chen Z. (2020). MicroRNA-21 contributes to renal cell carcinoma cell invasiveness and angiogenesis via the PDCD4/c-Jun (AP-1) signalling pathway. Int. J. Oncol..

[128] Zhang S., Zhang R., Xu R., Shang J., He H., Yang Q. (2020). MicroRNA-574-5p in gastric cancer cells promotes angiogenesis by targeting protein tyrosine phosphatase non-receptor type 3 (PTPN3). Gene.

[129] Shang D., Xie C., Hu J., Tan J., Yuan Y., Liu Z., Yang Z. (2020). Pancreatic cancer cell–derived exosomal microRNA-27a promotes angiogenesis of human microvascular endothelial cells in pancreatic cancer via BTG2. J. Cell. Mol. Med..

[130] Deng T., Zhang H., Yang H., Wang H., Bai M., Sun W., Wang X., Si Y., Ning T., Zhang L. (2020). Exosome miR-155 Derived from Gastric Carcinoma Promotes Angiogenesis by Targeting the c-MYB/VEGF Axis of Endothelial Cells. Mol. Ther.-Nucleic Acids.

[131] Shang A., Wang X., Gu C., Liu W., Sun J., Zeng B., Chen C., Ji P., Wu J., Quan W. (2020). Exosomal miR-183-5p promotes angiogenesis in colorectal cancer by regulation of FOXO1. Aging.

[132] Kim D.H., Park S., Kim H., Choi Y.J., Kim S.Y., Sung K.J., Sung Y.H., Choi C.-M., Yun M., Yi Y.-S. (2020). Tumor-derived exosomal miR-619-5p promotes tumor angiogenesis and metastasis through the inhibition of RCAN1.4. Cancer Lett..

[133] Zhang P., Ha M., Li L., Huang X., Liu C. (2020). MicroRNA-3064-5p sponged by MALAT1 suppresses angiogenesis in human hepatocellular carcinoma by targeting the FOXA1/CD24/Src pathway. FASEB J..

[134] Xu D., Yang F., Wu K., Xu X., Zeng K., An Y., Xu F., Xun J., Lv X., Zhang X. (2020). Lost miR-141 and upregulated TM4SF1 expressions associate with poor prognosis of pancreatic cancer: Regulation of EMT and angiogenesis by miR-141 and TM4SF1 via AKT. Cancer Biol. Ther..

[135] Liu H., Chen Y., Li Y., Li C., Qin T., Bai M., Zhang Z., Jia R., Su Y., Wang C. (2019). miR-195 suppresses metastasis and angiogenesis of squamous cell lung cancer by inhibiting the expression of VEGF. Mol. Med. Rep..

[136] Niu J., Li Z., Li F. (2019). Overexpressed microRNA-136 works as a cancer suppressor in gallbladder cancer through suppression of JNK signaling pathway via inhibition of MAP2K4. Am. J. Physiol. Liver Physiol..

[137] Cao J., Li L., Han X., Cheng H., Chen W., Qi K., Chen C., Wu Q., Niu M., Zeng L. (2019). miR-302 cluster inhibits angiogenesis and growth of K562 leukemia cells by targeting VEGFA. OncoTargets Ther..

[138] Li Y., Cai B., Shen L., Dong Y., Lu Q., Sun S., Liu S., Ma S., Ma P.X., Chen J. (2017). MiRNA-29b suppresses tumor growth through simultaneously inhibiting angiogenesis and tumorigenesis by targeting Akt3. Cancer Lett..

[139] Lu Y., Yu S.-S., Zong M., Fan S.-S., Lu T.-B., Gong R.-H., Sun L.-S., Fan L.-Y. (2017). Glucose-6-Phosphate Isomerase (G6PI) Mediates Hypoxia-Induced Angiogenesis in Rheumatoid Arthritis. Sci. Rep..

[140] Xie M., Dart D.A., Guo T., Xing X.-F., Cheng X.-J., Du H., Jiang W.G., Wen X.-Z., Ji J.-F. (2017). MicroRNA-1 acts as a tumor suppressor microRNA by inhibiting angiogenesis-related growth factors in human gastric cancer. Gastric Cancer.

[141] Lin Z.-Y., Chen G., Zhang Y.-Q., He H.-C., Liang Y.-X., Ye J.-H., Liang Y.-K., Mo R.-J., Lu J.-M., Zhuo Y.-J. (2017). MicroRNA-30d promotes angiogenesis and tumor growth via MYPT1/c-JUN/VEGFA pathway and predicts aggressive outcome in prostate cancer. Mol. Cancer.

[142] Seyfried T.N., Huysentruyt L.C. (2013). On the Origin of Cancer Metastasis. Crit. Rev. Oncog..

[143] Campbell K. (2018). Contribution of epithelial-mesenchymal transitions to organogenesis and cancer metastasis. Curr. Opin. Cell Biol..

[144] Friedl P., Mayor R. (2017). Tuning Collective Cell Migration by Cell–Cell Junction Regulation. Cold Spring Harb. Perspect. Biol..

[145] Janiszewska M., Primi M.C., Izard T. (2020). Cell adhesion in cancer: Beyond the migration of single cells. J. Biol. Chem..

[146] Yeung K.T., Yang J. (2017). Epithelial-mesenchymal transition in tumor metastasis. Mol. Oncol..

[147] Alberts B., Johnson A., Lewis J., Raff M., Roberts K., Walter P. (2020). Cell junctions. Molecular Biology of the Cell.

[148] Wu J.-I., Wang L.-H. (2019). Emerging roles of gap junction proteins connexins in cancer metastasis, chemoresistance and clinical application. J. Biomed. Sci..

[149] Zihni C., Balda M.S., Matter K. (2014). Signalling at tight junctions during epithelial differentiation and microbial pathogenesis. J. Cell Sci..

[150] Knights A., Funnell A.P.W., Crossley M., Pearson R.C.M. (2012). Holding Tight: Cell Junctions and Cancer Spread. Trends Cancer Res..

[151] Yu Y., Elble R.C. (2016). Homeostatic Signaling by Cell–Cell Junctions and Its Dysregulation during Cancer Progression. J. Clin. Med..

[152] Chen Q., Boire A., Jin X., Valiente M., Er E.E., Lopez-Soto A., Jacob L.S., Patwa R., Shah H., Xu K. (2016). Carcinoma—Astrocyte gap junctions promote brain metastasis by cGAMP transfer. Nature.

[153] Liu M., Yang J., Zhang Y., Zhou Z., Cui X., Zhang L., Fung K.-M., Zheng W., Allard F.D., Yee E.U. (2018). ZIP4 Promotes Pancreatic Cancer Progression by Repressing ZO-1 and Claudin-1 through a ZEB1-Dependent Transcriptional Mechanism. Clin. Cancer Res..

[154] Todd M.C., Petty H.M., King J., Marshall B.N.P., Sheller R.A., Cuevas M.E. (2015). Overexpression and delocalization of claudin-3 protein in MCF-7 and MDA-MB-415 breast cancer cell lines. Oncol. Lett..

[155] Lin C., Xin S., Qin X., Li H., Lin L., You Y. (2016). Zoledronic acid suppresses metastasis of esophageal squamous cell carcinoma cells through upregulating the tight junction protein occludin. Cytotechnology.

[156] Sinkevicius K.W., Bellaria K.J., Barrios J., Pessina P., Gupta M., Brainson C.F., Bronson R.T., Kim C.F. (2018). E-Cadherin Loss Accelerates Tumor Progression and Metastasis in a Mouse Model of Lung Adenocarcinoma. Am. J. Respir. Cell Mol. Biol..

[157] Han J., Xie C., Pei T., Wang J., Lan Y., Huang K., Cui Y., Wang F., Zhang J., Pan S. (2017). Deregulated AJAP1/β-catenin/ZEB1 signaling promotes hepatocellular carcinoma carcinogenesis and metastasis. Cell Death Dis..

[158] Lee S.-H., Min J.-K. (2018). Abstract 1082: Loss of desmoglein 2 promotes tumor growth and progression through the activation of Src and facilitates the internalization of EGFR in biliary tract carcinoma cells. Cancer Res..

[159] Li D., Song H., Mei H., Fang E., Wang X., Yang F., Li H., Chen Y., Huang K., Zheng L. (2018). Armadillo repeat containing 12 promotes neuroblastoma progression through interaction with retinoblastoma binding protein 4. Nat. Commun..

[160] Sang Y., Sun L., Wu Y., Yuan W., Liu Y., Li S.-W. (2019). Histone deacetylase 7 inhibits plakoglobin expression to promote lung cancer cell growth and metastasis. Int. J. Oncol..

[161] Cui T., Yang L., Ma Y., Petersen I., Chen Y. (2019). Desmocollin 3 has a tumor suppressive activity through inhibition of AKT pathway in colorectal cancer. Exp. Cell Res..

[162] Croset M., Pantano F., Kan C.W.S., Bonnelye E., Descotes F., Alix-Panabières C., Lecellier C.-H., Bachelier R., Allioli N., Hong S.-S. (2018). miRNA-30 Family Members Inhibit Breast Cancer Invasion, Osteomimicry, and Bone Destruction by Directly Targeting Multiple Bone Metastasis–Associated Genes. Cancer Res..

[163] McAnena P., Tanriverdi K., Curran C., Gilligan K., Freedman J.E., Brown J.A.L., Kerin M.J. (2019). Circulating microRNAs miR-331 and miR-195 differentiate local luminal a from metastatic breast cancer. BMC Cancer.

[164] Luo C., Yin D., Zhan H., Borjigin U., Li C., Zhou Z., Hu Z., Wang P., Sun Q., Fan J. (2018). microRNA-501-3p suppresses metastasis and progression of hepatocellular carcinoma through targeting LIN7A. Cell Death Dis..

[165] Hong B.S., Ryu H.S., Kim N., Kim J., Lee E., Moon H., Kim K.H., Jin M.-S., Kwon N.H., Kim S. (2019). Tumor suppressor microRNA-204-5p regulates growth, metastasis, and immune microenvironment remodeling in breast cancer. Cancer Res..

[166] Fu X.-T., Shi Y.-H., Zhou J., Peng Y.-F., Liu W.-R., Shi G.-M., Gao Q., Wang X.-Y., Song K., Fan J. (2018). MicroRNA-30a suppresses autophagy-mediated anoikis resistance and metastasis in hepatocellular carcinoma. Cancer Lett..

[167] Zhang X., Sai B., Wang F., Wang L., Wang Y., Zheng L., Li G., Tang J., Xiang J. (2019). Hypoxic BMSC-derived exosomal miRNAs promote metastasis of lung cancer cells via STAT3-induced EMT. Mol. Cancer.

[168] Colden M., Dar A.A., Saini S., Dahiya P.V., Shahryari V., Yamamura S., Tanaka Y., Stein G., Dahiya R., Majid S. (2018). MicroRNA-466 inhibits tumor growth and bone metastasis in prostate cancer by direct regulation of osteogenic transcription factor RUNX2. Cell Death Dis..

[169] Lohcharoenkal W., Das Mahapatra K., Pasquali L., Crudden C., Kular L., Ulum Y.Z.A., Zhang L., Landén N.X., Girnita L., Jagodic M. (2018). Genome-Wide Screen for MicroRNAs Reveals a Role for miR-203 in Melanoma Metastasis. J. Investig. Dermatol..

[170] Fang J., Zhang Z., Shang L., Luo Y., Lin Y., Yuan Y., Zhuang S. (2018). Hepatoma cell-secreted exosomal microRNA-103 increases vascular permeability and promotes metastasis by targeting junction proteins. Hepatology.

[171] Ke J., Shao W., Jiang Y., Xu J., Li F., Qin J. (2018). MicroRNA-103 regulates tumorigenesis in colorectal cancer by targeting ZO-1. Mol. Med. Rep..

[172] De Oliveira J.G., Marques J.H.D.M., Lacerda J.Z., Ferreira L.C., Coelho M.M.C., Zuccari D.A.P.D.C. (2019). Melatonin down-regulates microRNA-10a and decreases invasion and migration of triple-negative breast cancer cells. Melatonin Res..

[173] Wang H., Tan Z., Hu H., Liu H., Wu T., Zheng C., Wang X., Luo Z., Wang J., Liu S. (2019). microRNA-21 promotes breast cancer proliferation and metastasis by targeting LZTFL1. BMC Cancer.

[174] Zhang R., Shi H., Ren F., Feng W., Cao Y., Li G., Liu Z., Ji P., Zhang M. (2019). MicroRNA-338-3p suppresses ovarian cancer cells growth and metastasis: Implication of Wnt/catenin beta and MEK/ERK signaling pathways. J. Exp. Clin. Cancer Res..

[175] Grisard E., Coan M., Cesaratto L., Rigo I., Zandonà L., Paulitti A., Andreuzzi E., Vinciguerra G.L.R., Poletto E., Del Ben F. (2019). Sleeping beauty genetic screen identifies miR-23b::BTBD7 gene interaction as crucial for colorectal cancer metastasis. EBioMedicine.

[176] Niu J., Xue A., Chi Y., Xue J., Wang W., Zhao Z., Fan M., Yang C.H., Shao Z.-M., Pfeffer L. (2016). Induction of miRNA-181a by genotoxic treatments promotes chemotherapeutic resistance and metastasis in breast cancer. Oncogene.

[177] Xu Q., Liu X., Liu Z., Zhou Z., Wang Y., Tu J., Li L., Bao H., Yang L., Tu K. (2017). MicroRNA-1296 inhibits metastasis and epithelial-mesenchymal transition of hepatocellular carcinoma by targeting SRPK1-mediated PI3K/AKT pathway. Mol. Cancer.

[178] Gimple R.C., Wang X. (2019). RAS: Striking at the Core of the Oncogenic Circuitry. Front. Oncol..

[179] David C.J., Chen M., Assanah M., Canoll P., Manley J. (2009). HnRNP proteins controlled by c-Myc deregulate pyruvate kinase mRNA splicing in cancer. Nature.

[180] Shim H., Dolde C., Lewis B.C., Wu C.-S., Dang G., Jungmann R.A., Dalla-Favera R., Dang C.V. (1997). c-Myc transactivation of LDH-A: Implications for tumor metabolism and growth. Proc. Natl. Acad. Sci. USA.

[181] Wise D., DeBerardinis R.J., Mancuso A., Sayed N., Zhang X.-Y., Pfeiffer H.K., Nissim I., Daikhin E., Yudkoff M., McMahon S.B. (2008). Myc regulates a transcriptional program that stimulates mitochondrial glutaminolysis and leads to glutamine addiction. Proc. Natl. Acad. Sci. USA.

[182] Schwartzenberg-Bar-Yoseph F., Armoni M., Karnieli E. (2004). The Tumor Suppressor p53 Down-Regulates Glucose Transporters GLUT1 and GLUT4 Gene Expression. Cancer Res..

[183] Wang L., Xiong H., Wu F., Zhang Y., Wang J., Zhao L., Guo X., Chang L.-J., Zhang Y., You M.J. (2014). Hexokinase 2-Mediated Warburg Effect Is Required for PTEN- and p53-Deficiency-Driven Prostate Cancer Growth. Cell Rep..

[184] Liu J., Zhang C., Zhao Y., Yue X., Wu H., Huang S., Chen J., Tomsky K., Xie H., Khella C.A. (2017). Parkin targets HIF-1α for ubiquitination and degradation to inhibit breast tumor progression. Nat. Commun..

[185] Wieman H.L., Wofford J.A., Rathmell J.C. (2007). Cytokine Stimulation Promotes Glucose Uptake via Phosphatidylinositol-3 Kinase/Akt Regulation of Glut1 Activity and Trafficking. Mol. Biol. Cell.

[186] Wu N., Zheng B., Shaywitz A., Dagon Y., Tower C., Bellinger G., Shen C.-H., Wen J., Asara J., McGraw T.E. (2013). AMPK-Dependent Degradation of TXNIP upon Energy Stress Leads to Enhanced Glucose Uptake via GLUT1. Mol. Cell.

[187] Chen C., Pore N., Behrooz A., Ismail-Beigi F., Maity A. (2001). Regulation of glut1 mRNA by hypoxia-inducible factor-1 Interaction between H-ras and hypoxia. J. Biol. Chem..

[188] He G., Jiang Y., Zhang B., Wu G. (2014). The effect of HIF-1alpha on glucose metabolism, growth and apoptosis of pancreatic cancerous cells. Asia Pac. J. Clin. Nutr..

[189] Iyer N.V., Kotch L.E., Agani F., Leung S.W., Laughner E., Wenger R.H., Gassmann M., Gearhart J.D., Lawler A.M., Aimee Y.Y. (1998). Cellular and developmental control of O2 homeostasis by hypoxia-inducible factor 1α. Genes Dev..

[190] Mahon P.C., Hirota K., Semenza G.L. (2001). FIH-1: A novel protein that interacts with HIF-1α and VHL to mediate repression of HIF-1 transcriptional activity. Genes Dev..

[191] Maxwell P., Wiesener M.S., Chang G.-W., Clifford S.C., Vaux E.C., Cockman M., Wykoff C.C., Pugh C., Maher E., Ratcliffe P. (1999). The tumour suppressor protein VHL targets hypoxia-inducible factors for oxygen-dependent proteolysis. Nature.

[192] Heiden M.G.V., Cantley L.C., Thompson C.B. (2009). Understanding the Warburg Effect: The Metabolic Requirements of Cell Proliferation. Science.

[193] Warburg O., Wind F., Negelein E. (1927). The metabolism of tumors in the body. J. Gen. Physiol..

[194] Soga T. (2013). Cancer metabolism: Key players in metabolic reprogramming. Cancer Sci..

[195] Santos C.R., Schulze A. (2012). Lipid metabolism in cancer. FEBS J..

[196] Liu Q., Luo Q., Halim A., Song G. (2017). Targeting lipid metabolism of cancer cells: A promising therapeutic strategy for cancer. Cancer Lett..

[197] Tsun Z.-Y., Possemato R. (2015). Amino acid management in cancer. Semin. Cell Dev. Biol..

[198] DeBerardinis R.J., Cheng T. (2010). Q’s next: The diverse functions of glutamine in metabolism, cell biology and cancer. Oncogene.

[199] Zhao X., Lu C., Chu W., Zhang B., Zhen Q., Wang R., Zhang Y., Li Z., Lv B., Li H. (2017). MicroRNA-124 suppresses proliferation and glycolysis in non–small cell lung cancer cells by targeting AKT–GLUT1/HKII. Tumor Biol..

[200] Zhu B., Cao X., Zhang W., Pan G., Yi Q., Zhong W., Yan D. (2019). MicroRNA-31-5p enhances the Warburg effect via targeting FIH. FASEB J..

[201] Zhu W., Huang Y., Pan Q., Xiang P., Xie N., Yu H. (2017). MicroRNA-98 Suppress Warburg Effect by Targeting HK2 in Colon Cancer Cells. Dig. Dis. Sci..

[202] Minami K., Taniguchi K., Sugito N., Kuranaga Y., Inamoto T., Takahara K., Takai T., Yoshikawa Y., Kiyama S., Akao Y. (2017). MiR-145 negatively regulates Warburg effect by silencing KLF4 and PTBP1 in bladder cancer cells. Oncotarget.

[203] Li B., He L., Zuo D., He W., Wang Y., Zhang Y., Liu W., Yuan Y. (2017). Mutual Regulation of MiR-199a-5p and HIF-1α Modulates the Warburg Effect in Hepatocellular Carcinoma. J. Cancer.

[204] Chen H., Gao S., Cheng C. (2018). MiR-323a-3p suppressed the glycolysis of osteosarcoma via targeting LDHA. Hum. Cell.

[205] Liu Z., Wang J., Li Y., Fan J., Chen L., Xu R. (2017). MicroRNA-153 regulates glutamine metabolism in glioblastoma through targeting glutaminase. Tumor Biol..

[206] Liu L., Wang Y., Bai R., Yang K., Tian Z. (2016). MiR-186 inhibited aerobic glycolysis in gastric cancer via HIF-1α regulation. Oncogenesis.

[207] Yan W., Wu X., Zhou W., Fong M.Y., Cao M., Liu J., Liu X., Chen C.-H., Fadare O., Pizzo D.P. (2018). Cancer-cell-secreted exosomal miR-105 promotes tumour growth through the MYC-dependent metabolic reprogramming of stromal cells. Nature.

[208] Kim J., Lee S., Lim S., Yu S., Ku G. (2018). microRNA 181a-5p Reprogramed Glucose and Lipid Metabolism in Non-Small Cell Lung Cancer. J. Cancer Sci. Clin. Oncol..

[209] Yang Y., Gabra M.B.I., Hanse E.A., Lowman X.H., Tran T.Q., Li H., Milman N., Liu J., Reid M., Locasale J.W. (2019). MiR-135 suppresses glycolysis and promotes pancreatic cancer cell adaptation to metabolic stress by targeting phosphofructokinase. Nat. Commun..

[210] Xu F., Yan J.-J., Gan Y., Chang Y., Wang H.-L., He X.-X., Zhao Q. (2019). miR-885-5p Negatively Regulates Warburg Effect by Silencing Hexokinase 2 in Liver Cancer. Mol. Ther.-Nucleic Acids.

[211] Rupaimoole R., Calin G., Lopez-Berestein G., Sood A.K. (2016). miRNA Deregulation in Cancer Cells and the Tumor Microenvironment. Cancer Discov..

[212] Soon P., Kiaris H. (2013). MicroRNAs in the tumour microenvironment: Big role for small players. Endocr.-Relat. Cancer.

[213] Yang X., Li Y., Zou L., Zhu Z. (2019). Role of Exosomes in Crosstalk between Cancer-Associated Fibroblasts and Cancer Cells. Front. Oncol..

[214] Sun L., Xu K., Cui J., Yuan D., Zou B., Li J., Liu J., Li K., Meng Z., Zhang B. (2019). Cancer-associated fibroblast-derived exosomal miR-382-5p promotes the migration and invasion of oral squamous cell carcinoma. Oncol. Rep..

[215] Li Y.-Y., Tao Y.-W., Gao S., Li P., Zheng J.-M., Zhang S.-E., Liang J., Zhang Y. (2018). Cancer-associated fibroblasts contribute to oral cancer cells proliferation and metastasis via exosome-mediated paracrine miR-34a-5p. EBioMedicine.

[216] Xu G., Zhang B., Ye J., Cao S., Shi J., Zhao Y., Wang Y., Sang J., Yao Y., Guan W. (2019). Exosomal miRNA-139 in cancer-associated fibroblasts inhibits gastric cancer progression by repressing MMP11 expression. Int. J. Biol. Sci..

[217] Wang R., Sun Y., Yu W., Yan Y., Qiao M., Jiang R., Guan W., Wang L. (2019). Downregulation of miRNA-214 in cancer-associated fibroblasts contributes to migration and invasion of gastric cancer cells through targeting FGF9 and inducing EMT. J. Exp. Clin. Cancer Res..

[218] Zhang Y., Cai H., Chen S., Sun D., Zhang D., He Y. (2019). Exosomal transfer of miR-124 inhibits normal fibroblasts to cancer-associated fibroblasts transition by targeting sphingosine kinase 1 in ovarian cancer. J. Cell. Biochem..

[219] Zhou Y., Zhong J.-H., Gong F.-S., Xiao J. (2019). MiR-141-3p suppresses gastric cancer induced transition of normal fibroblast and BMSC to cancer-associated fibroblasts via targeting STAT4. Exp. Mol. Pathol..

[220] Kim C.W., Oh E.-T., Kim J.M., Park J.-S., Lee D.H., Lee J.-S., Kim K.K., Park H.J. (2018). Hypoxia-induced microRNA-590-5p promotes colorectal cancer progression by modulating matrix metalloproteinase activity. Cancer Lett..

[221] Zheng H., Bi F.-R., Yang Y., Hong Y.-G., Ni J.-S., Ma L., Liu M.-H., Hao L.-Q., Zhou W.-P., Song L.-H. (2019). Downregulation of miR-196-5p Induced by Hypoxia Drives Tumorigenesis and Metastasis in Hepatocellular Carcinoma. Horm. Cancer.

[222] Ni J., Zhou S., Yuan W., Cen F., Yan Q. (2019). Mechanism of miR-210 involved in epithelial–mesenchymal transition of pancreatic cancer cells under hypoxia. J. Recept. Signal Transduct..

[223] Angel C.Z., Lynch S.M., Nesbitt H., McKenna M.M., Walsh C.P., McKenna D.J. (2020). miR-210 is induced by hypoxia and regulates neural cell adhesion molecule in prostate cells. J. Cell. Physiol..

[224] Zhou Y., Ren H., Dai B., Li J., Shang L., Huang J., Shi X. (2018). Hepatocellular carcinoma-derived exosomal miRNA-21 contributes to tumor progression by converting hepatocyte stellate cells to cancer-associated fibroblasts. J. Exp. Clin. Cancer Res..

[225] Wang X., Qin X., Yan M., Shi J., Xu Q., Li Z., Yang W., Zhang J., Chen W. (2019). Loss of exosomal miR-3188 in cancer-associated fibroblasts contributes to HNC progression. J. Exp. Clin. Cancer Res..

[226] Medeiros M., Ribeiro A., Lupi L.A., Romualdo G.R., Pinhal D., Chuffa L.G.D.A., Delella F.K. (2019). Mimicking the tumor microenvironment: Fibroblasts reduce miR-29b expression and increase the motility of ovarian cancer cells in a co-culture model. Biochem. Biophys. Res. Commun..

[227] Li Y., Zhao Z., Liu W., Li X. (2020). SNHG3 Functions as miRNA Sponge to Promote Breast Cancer Cells Growth through the Metabolic Reprogramming. Appl. Biochem. Biotechnol..

[228] Vu L.T., Peng B., Zhang D.X., Ma V., Mathey-Andrews C.A., Lam C.K., Kiomourtzis T., Jin J., McReynolds L., Huang L. (2019). Tumor-secreted extracellular vesicles promote the activation of cancer-associated fibroblasts via the transfer of microRNA-125b. J. Extracell. Vesicles.

[229] Wang J., Guan X., Zhang Y., Ge S., Zhang L., Li H., Wang X., Liu R., Ning T., Deng T. (2018). Exosomal miR-27a Derived from Gastric Cancer Cells Regulates the Transformation of Fibroblasts into Cancer-Associated Fibroblasts. Cell. Physiol. Biochem..

[230] Fang T., Lv H., Lv G., Li T., Wang C., Han Q., Yu L., Su B., Guo L., Huang S. (2018). Tumor-derived exosomal miR-1247-3p induces cancer-associated fibroblast activation to foster lung metastasis of liver cancer. Nat. Commun..

[231] Qin X., Guo H., Wang X., Zhu X., Yan M., Wang X., Xu Q., Shi J., Lu E., Chen W. (2019). Exosomal miR-196a derived from cancer-associated fibroblasts confers cisplatin resistance in head and neck cancer through targeting CDKN1B and ING5. Genome Biol..

[232] Zhang Q., Zhang J., Fu Z., Dong L., Tang Y., Xu C., Wang H., Zhang T., Wu Y., Dong C. (2019). Hypoxia-induced microRNA-10b-3p promotes esophageal squamous cell carcinoma growth and metastasis by targeting TSGA10. Aging.

[233] McCoach C.E., Bivona T.G. (2018). The evolving understanding of immunoediting and the clinical impact of immune escape. J. Thorac. Dis..

[234] Eichmüller S., Osen W., Mandelboim O., Seliger B. (2017). Immune Modulatory microRNAs Involved in Tumor Attack and Tumor Immune Escape. J. Natl. Cancer Inst..

[235] Syed S.N., Frank A.-C., Raue R., Brüne B. (2019). MicroRNA—A Tumor Trojan Horse for Tumor-Associated Macrophages. Cells.

[236] Chen C., Liu J.-M., Luo Y.-P. (2019). MicroRNAs in tumor immunity: Functional regulation in tumor-associated macrophages. J. Zhejiang Univ. Sci. B.

[237] Omar H.A., El-Serafi A.T., Hersi F., Arafa E.A., Zaher D.M., Madkour M., Arab H.H., Tolba M.F. (2019). Immunomodulatory MicroRNAs in cancer: Targeting immune checkpoints and the tumor microenvironment. FEBS J..

[238] Sica A., Mantovani A. (2012). Macrophage plasticity and polarization: In Vivo veritas. J. Clin. Investig..

[239] Sahraei M., Chaube B., Liu Y., Sun J., Kaplan A., Price N., Ding W., Oyaghire S., García-Milian R., Mehta S. (2019). Suppressing miR-21 activity in tumor-associated macrophages promotes an antitumor immune response. J. Clin. Investig..

[240] Cooks T., Pateras I.S., Jenkins L.M., Patel K.M., Robles A.I., Morris J., Forshew T., Appella E., Gorgoulis V., Harris C.C. (2018). Mutant p53 cancers reprogram macrophages to tumor supporting macrophages via exosomal miR-1246. Nat. Commun..

[241] Zhou J., Li X., Wu X., Zhang T., Zhu Q., Wang X., Wang H., Wang K., Lin Y., Wang X. (2018). Exosomes Released from Tumor-Associated Macrophages Transfer miRNAs That Induce a Treg/Th17 Cell Imbalance in Epithelial Ovarian Cancer. Cancer Immunol. Res..

[242] Ke M., Zhang Z., Cong L., Zhao S., Li Y., Wang X., Lv Y., Zhu Y., Dong J. (2019). MicroRNA-148b-colony-stimulating factor-1 signaling-induced tumor-associated macrophage infiltration promotes hepatocellular carcinoma metastasis. Biomed. Pharmacother..

[243] Ren W., Zhang X., Li W., Feng Q., Feng H., Tong Y., Rong H., Wang W., Zhang D., Zhang Z. (2019). Exosomal miRNA-107 induces myeloid-derived suppressor cell expansion in gastric cancer. Cancer Manag. Res..

[244] Ding L., Li Q., Chakrabarti J., Muñoz A., Faure-Kumar E., Ocadiz-Ruiz J.R., Razumilava N., Zhang G., Hayes M.H., A Sontz R. (2020). MiR130b from Schlafen4+ MDSCs stimulates epithelial proliferation and correlates with preneoplastic changes prior to gastric cancer. Gut.

[245] Huber V., Vallacchi V., Fleming V., Hu X., Cova A., Dugo M., Shahaj E., Sulsenti R., Vergani E., Filipazzi P. (2018). Tumor-derived microRNAs induce myeloid suppressor cells and predict immunotherapy resistance in melanoma. J. Clin. Investig..

[246] Guo X., Qiu W., Liu Q., Qian M., Wang S., Zhang Z., Gao X., Chen Z., Xue H., Li G. (2018). Immunosuppressive effects of hypoxia-induced glioma exosomes through myeloid-derived suppressor cells via the miR-10a/Rora and miR-21/Pten Pathways. Oncogene.

[247] Tang S., Fu H., Xu Q., Zhou Y. (2019). miR-20a regulates sensitivity of colorectal cancer cells to NK cells by targeting MICA. Biosci. Rep..

[248] Zhou X., Liu S., Liu J., Zhang Z., Mao X., Zhou H. (2020). MicroRNA-130a enhances the killing ability of natural killer cells against non-small cell lung cancer cells by targeting signal transducers and activators of transcription 3. Biochem. Biophys. Res. Commun..

[249] Yang Q., Li J., Hu Y., Tang X., Yu L., Dong L., Chen D. (2019). MiR-218-5p Suppresses the Killing Effect of Natural Killer Cell to Lung Adenocarcinoma by Targeting SHMT1. Yonsei Med. J..

[250] Nanbakhsh A., Srinivasamani A., Holzhauer S., Riese M.J., Zheng Y., Wang D., Burns R., Reimer M.H., Rao S., Lemke A. (2019). Mirc11 Disrupts Inflammatory but Not Cytotoxic Responses of NK Cells. Cancer Immunol. Res..

[251] Chen E.-B., Zhou Z.-J., Xiao K., Zhu G.-Q., Yang Y., Wang B., Zhou S.-L., Chen Q., Yin D., Wang Z. (2019). The miR-561-5p/CX3CL1 Signaling Axis Regulates Pulmonary Metastasis in Hepatocellular Carcinoma Involving CX3CR1+ Natural Killer Cells Infiltration. Theranostics.

[252] Ye S.-B., Zhang H., Cai T.-T., Liu Y.-N., Ni J.-J., He J., Peng J.-Y., Chen Q.-Y., Mo H.-Y., Cui J. (2016). Exosomal miR-24-3p impedes T-cell function by targetingFGF11and serves as a potential prognostic biomarker for nasopharyngeal carcinoma. J. Pathol..

[253] Zhao M., Liu Q., Liu W., Zhou H., Zang X., Lu J. (2019). MicroRNA-140 suppresses Helicobacter pylori-positive gastric cancer growth by enhancing the antitumor immune response. Mol. Med. Rep..

[254] Zhang M., Gao D., Shi Y., Wang Y., Joshi R., Yu Q., Liu D., Alotaibi F., Zhang Y., Wang H. (2019). miR-149-3p reverses CD8 + T-cell exhaustion by reducing inhibitory receptors and promoting cytokine secretion in breast cancer cells. Open Biol..

[255] Lou Q., Liu R.-X., Yang X., Li W., Huang L., Wei L., Tan H., Xiang N., Chan K., Chen J. (2019). miR-448 targets IDO1 and regulates CD8+ T cell response in human colon cancer. J. Immunother. Cancer.

[256] Yuan C., Xiang L., Bai R., Cao K., Gao Y., Jiang X., Zhang N., Gong Y., Xie C. (2019). MiR-195 restrains lung adenocarcinoma by regulating CD4+ T cell activation via the CCDC88C/Wnt signaling pathway: A study based on the Cancer Genome Atlas (TCGA), Gene Expression Omnibus (GEO) and bioinformatic analysis. Ann. Transl. Med..

[257] Liu J. (2018). The dualistic origin of human tumors. Semin. Cancer Biol..

[258] Mirzayans R., Andrais B., Murray D. (2018). Roles of Polyploid/Multinucleated Giant Cancer Cells in Metastasis and Disease Relapse Following Anticancer Treatment. Cancers.

[259] Mirzayans R., Murray D. (2020). Intratumor Heterogeneity and Therapy Resistance: Contributions of Dormancy, Apoptosis Reversal (Anastasis) and Cell Fusion to Disease Recurrence. Int. J. Mol. Sci..

[260] War A.R., Dang K., Jiang S., Xiao Z., Miao Z., Yang T., Li Y., Qian A. (2020). Role of cancer stem cells in the development of giant cell tumor of bone. Cancer Cell Int..

[261] Xu Y., So C., Lam H.-M., Fung M.-C., Tsang S.-Y. (2018). Apoptosis Reversal Promotes Cancer Stem Cell-Like Cell Formation. Neoplasia.

[262] Zaitceva V., Kopeina G., Zhivotovsky B. (2021). Anastasis: Return Journey from Cell Death. Cancers.

[263] Yao Q., Chen Y., Zhou X., Yao Q., Chen Y., Zhou X. (2019). The roles of microRNAs in epigenetic regulation. Curr. Opin. Chem. Biol..

[264] Arif K.M.T., Elliott E.K., Haupt L.M., Griffiths L.R. (2020). Regulatory Mechanisms of Epigenetic miRNA Relationships in Human Cancer and Potential as Therapeutic Targets. Cancers.

